# AI can outperform humans in predicting correlations between personality items

**DOI:** 10.1038/s44271-025-00205-w

**Published:** 2025-02-12

**Authors:** Philipp Schoenegger, Spencer Greenberg, Alexander Grishin, Joshua Lewis, Lucius Caviola

**Affiliations:** 1 London School of Economics and Political Science, London, UK; 2 Spark Wave, New York, NY USA; 3 New York University, New York, NY USA; 4 University of Oxford, Oxford, UK

**Keywords:** Psychology, Information systems and information technology

## Abstract

We assess the abilities of both specialized deep neural networks, such as PersonalityMap, and general LLMs, including GPT-4o and Claude 3 Opus, in understanding human personality by predicting correlations between personality questionnaire items. All AI models outperform the vast majority of laypeople and academic experts. However, we can improve the accuracy of individual correlation predictions by taking the median prediction per group to produce a “wisdom of the crowds” estimate. Thus, we also compare the median predictions from laypeople, academic experts, GPT-4o/Claude 3 Opus, and PersonalityMap. Based on medians, PersonalityMap and academic experts surpass both LLMs and laypeople on most measures. These results suggest that while advanced LLMs make superior predictions compared to most individual humans, specialized models like PersonalityMap can match even expert group-level performance in domain-specific tasks. This underscores the capabilities of large language models while emphasizing the continued relevance of specialized systems as well as human experts for personality research.

## Abstract (web summary)

Specialized neural networks and general large language models outperform individual humans at predicting personality questionnaire item correlations, but specialized models and aggregated expert predictions achieve the highest accuracy on most measures.

## Introduction

Even though modern artificial intelligence (AI) is fundamentally distinct from humans and human intelligence, it is able to learn about human psychology. But how well do modern AI systems understand human personality? In this study, we test how accurately a set of AI models can predict the correlations between human personality questionnaire items. We test whether AI models are better at this than lay people or even psychology experts. Additionally, we compare the prediction capabilities of generalised large language models (LLMs; e.g., ChatGPT) and a specialised AI system (PersonalityMap—from https://personalitymap.io) trained specifically with empirical personality data, as well as other AI models.

Modern LLMs, due to the attention mechanism^1,2^ of the transformer architecture^3^, have shown strong performance in a large number of diverse domains, such as marketing^4^, teaching^5^, programming^6^, medicine^7,8^, and legal reasoning^9^. Perhaps the most impressive aspect of their performance is that a single machine learning model can excel at such a distinct set of tasks, often close to, at, or even above human level, with some models sometimes matching or even exceeding human experts. However, there remain numerous academic and practical use cases where more specialised deep neural networks, almost always significantly smaller and built on older architectures, continue to be used, like in the contexts of ultrasonography diagnosis^10^, materials science^11^, or athletics^12^. These specialised models often outperform due to context specific requirements, direct training on the relevant goal, and the ability to leverage high quality, proprietary data^13^.

For the purposes of this paper, we distinguish domain-specific (specialised) and domain-general (generalised) models. Both types of machine learning models share the feature that they are trained with a quite narrow objective such as predicting the next token. However, specialised models, as they are deployed, only take a very specific type of input (such as tabular data, genetic profiles, or a picture) and provide a specific output (such as a statistical value, a health risk profile, or a classification). Contrast this with generalised models like LLMs that, due to their architecture, can operate with a large number of inputs as well as outputs ranging from words to numbers and even images. Not only do LLMs enable a variety of modalities, they are also domain-general in that one can ask them about a puppy’s health condition, interest rate projections for Peru, or an interpretation of a regression output table, primarily because of their vast training data. This makes these models different from previous AI models, raising the question of how well generalised models (LLMs) perform compared to specialised models in a number of specific applications.

In this paper, we provide a direct test of this potential divergence between types of modern AI models. Specifically, in our preregistered analyses we test the ability of two frontier LLMs (GPT-4o and Claude 3 Opus), a specialised model (PersonalityMap), lay people, and academic experts to predict the relationship between two survey questions (called ‘items’). In non-preregistered analyses, we also test a second specialised model (SurveyBot3000) as well as run one of the frontier LLMs (GPT-4o) at different specifications. The items we test are drawn from the personality psychology literature, testing how well these machine learning models can understand the details of human personality. For example, we may ask the model or human participant to predict the relationship (as a correlation coefficient) between the following two items: “I seek to influence others” and “I enjoy interactions less than others”. The former may be related to Machiavellianism^14^, a manipulative personality trait of the dark triad^15^, while the second may be best understood as relating to introversion^16^, where those who score higher on introvert measures may agree with the statement above more frequently than those who do not. Predicting the relationship between these two items may be possible by direct recourse to the academic literature, though the specific pairs queried are at best only indirectly studied, like in this case^17^. Some parts of these relationships may also be gleaned by a casual understanding of human psychology or personal experiences. This makes this data an interesting test case, as generalised models may be able to grasp a large portion of these relationships directly, though specialised models, trained directly on these types of correlations, may be even more performant, though whether this is the case remains an open question that we test in this paper. For all these comparisons, we provide human lay and human expert comparisons to properly contextualise model performance in relative terms.

There is not much previous academic work that has compared lay people, experts, and machine predictions on the relationship between psychometric items from personality research. Some related work^18^ has looked at Random Forest predictions of personality traits based on written interview data. Their model inferred HEXACO personality traits with average correlations of between 0.31 and 0.39 depending on modelling choices. Recent work in the context of expert predictions of long-run RCT effects has found that while academic experts predict effect sizes of studies better than laypeople, they fail to pass simple benchmarks^19^, suggesting that humans generally struggle to predict outcomes of academic studies and results. In more recent relevant literature, Hommel & Arslan^20^ introduced a generalised AI model called SurveyBot3000, which is a fine-tune of the sentence transformer all-mpnet-base-v2. In their analysis of data drawn from Bainbridge et al.^21^, they show that their approach can accurately predict correlations between survey items. Similarly, Wulff & Mata^22^, using fine-tuned embedding models, ranging from the BERT-type MPNet to OpenAI’s text-embedding-3-large and others, also manage to reconstruct relations between internal consistency measures of different psychological measures and enable prediction of empirical relations between items as well as scales.

Moreover, our work fits into a broader literature on LLMs and personality more generally. This work has investigated various aspects of how AI models understand and interact with human personality. For instance, Peters and Matz^23^ demonstrated that LLMs like GPT-3.5 and GPT-4 can accurately infer individuals’ Big Five personality traits from social media posts, highlighting the potential of LLMs to analyze psychological dispositions. Zhang et al.^24^ evaluated LLMs’ ability to assess personality from asynchronous video interviews, finding that while LLMs can achieve validity comparable to task-specific AI models, they exhibit uneven performance across different traits. Other research has focused on LLMs’ capability to simulate personality-congruent responses and adaptively interact with humans^24,25^, as well as assessed the psychological profiles of LLMs themselves through psychometric inventories^26^, and outlined concerns about the generalizability of personality tests to AI models^27^. Jiang et al.^28^ further explored inducing specific personality traits in pre-trained language models, demonstrating controlled and verifiable behavior changes.

Despite these advancements, there remains a gap in the field’s understanding: it is still unclear how well AI models comprehend and predict the underlying relationships between personality questionnaire items. Accurately predicting such correlations is useful for validating scales, testing hypotheses, and gaining deeper insights into the structure of personality traits. While previous research has focused on LLMs’ ability to simulate personality-consistent behavior or infer traits from text, few studies have directly addressed their ability to predict the correlations between individual personality items, which is central to psychometric research and practical research applications. By focusing on this specific task, our work fills a critical gap, showing how well frontier LLMs and specialised models can perform in comparison to human experts and laypeople.

There are many reasons that it can be useful to understand the relationships between self-reported personality items, including to test hypotheses (e.g., that people with anxiety also often have depression), to develop scales (e.g., to identify items that could help measure a trait such as narcissism), and to generate new hypotheses (e.g., by exploring what items are predictive reporting being unhappy with their relationships). For these same reasons, it can be useful to accurately predict the correlations between personality items. Additionally, such predictions of correlations are much faster to make than running studies to measure those correlations, so sufficiently accurate correlation predictions could facilitate rapid research—allowing the data collection (to confirm those predictions on real people) to be pushed back later at the end of the research process.

AI systems capable of reliably predicting these correlations could automate and streamline psychometric assessments, enhancing the development of personality scales and hypothesis testing. If general models like LLMs could achieve high accuracy, they might enable applications in hiring, healthcare, and personalized marketing, reducing reliance on extensive questionnaires or expert input while adapting quickly to new contexts.

In this paper, we investigate how accurately AI models can predict correlations between personality items compared to humans. We address this inquiry through two distinct approaches—individual and aggregate—each corresponding to a specific research question. First, how do *individual* AI models (LLMs and specialized deep neural networks) compare to *individual* humans (both laypeople and academic experts)? Second, how do AI and human predictions compare when their estimates are *aggregated* using median values for each item correlation? For each type of question, we conduct several preregistered analyses. This allows us to better understand the distinct capabilities and potential applications of AI and human approaches.

Our first research question is about how machine learning model approaches perform in the distribution of individual human predictions. This research question is important primarily for a head-to-head comparison between AI models and humans. For example, in academic research one may want to draw on correlations between items to help theory-building or experiment design. When these relationships are not yet studied (or may be the object of the study in question), researchers may use expert predictions as a stand-in for early hypothesis generation. Our individual analyses query whether models could be used instead of humans for tasks like this. To test this specifically, we approach it in two ways. First, we test whether the machine learning model approaches have a better or worse average error (over all predicted items) than the median lay person and the median expert (ranked based on the average error for the subset of questions they answered). Formally, we test the preregistered null hypothesis:

*Null Hypothesis 1a: The machine approaches do not outperform or underperform the median percentile rank of lay humans and experts*.

Second, we also directly compare each individual’s predictions one-to-one with an AI’s to investigate whether machine learning models beat most humans on the questions that they answered. That is, for each human, we count each AI approach as having “won” if it has a lower average error than the human in more than half of the items that the humans made estimates for. Note, because this comparison is with the median error, human populations do not benefit from error cancellation from aggregation, as they would if we took the error of the median estimate as we do in the second research question. Formally, we test the preregistered null hypothesis:

*Null Hypothesis 1b: The machine approaches do not win more or less than 50% of individual comparisons to lay humans or experts*.

These two hypotheses allow us to test the AI approaches on an individual level, i.e., how they would perform in a human context where a single model is introduced and pitted against individual humans (both lay people and academic experts). This approach might provide results most relevant to applied contexts in which decisionmakers must choose between relying on a single human judge or a single AI model. For example, some applied uses of personality research, such as automated personality assessment, may thus be able to primarily or exclusively rely on AI predictions as opposed to human expert output. Based on previous work, one may expect AI models to outperform the vast majority of lay people, while a clear directional prediction with respect to experts is less easily made.

We would like to point out that the wording of NH1b differs from our preregistered wording. This is because we made an error, and our preregistered wording was almost indistinguishable from NH1a. The preregistered analysis remains wholly unchanged, in that there are no deviations from our protocol. We have made this change solely to improve comprehensibility of our preregistered hypothesis and avoid misunderstandings.

Our second set of questions, aimed at our second research question, compares the accuracy of all the approaches by taking the median prediction for each item within each condition before comparison to arrive at an aggregate prediction. This set of analyses aims to give the AI approaches a tougher comparison to humans. While AI models might beat *individual* humans, even experts, aggregated human forecasts may prove a more difficult challenge as they draw on the distributed knowledge that a diverse group of humans inevitably possesses. In a sense, this allows us to test not whether individual AI queries can replace queries to individual experts, but whether these systems may be used as full stand-alone replacements to human expert uses too. After all, if even aggregations of expert opinions cannot provide better estimations of relationships, this might open up a whole host of applications across industries. To test whether this is indeed the case, we analyse the differences in prediction error, prediction correlation, and bucketised prediction error between the conditions. These individual scores indicate how well the different approaches work as an aggregate. We test the following three preregistered null hypotheses.

*Null Hypothesis 2a: There is no difference in prediction error between the conditions*.

*Null Hypothesis 2b: There is no difference in prediction correlation between the conditions*.

*Null Hypothesis 2c: There is no difference in bucketised prediction error between the Conditions*.

This set of hypotheses lets us test the predictive capability of all approaches as an aggregate across three plausible types of comparisons. Specifically, this allows us to benefit from the error-cancellation that aggregating individual predictions brings with it, called the ‘wisdom of the crowd’ effect: This ‘wisdom of the crowd’ effect has been documented in both human^29^ and machine learning model^30,31^ contexts. In our case, we aggregate across 3 items for the LLM conditions and over at least 16 predictions in each of the human conditions on each item. We then test these aggregate values on a set of three distinct outcome variables. Overall, this approach provides general results with respect to the capabilities of a given model or human population, with the potential of more wide-ranging applications in both research and industry. Previous work on aggregated AI predictions^31^ might suggest that the human conditions would perform better against AI models compared to our earlier set of comparisons.

## Methods

Our study collects data from five different sources for our preregistered analyses. All different sources provide correlations between sets of item pairs drawn from the personality psychology literature First, we collect predictions from a layperson population recruited via Positly, an online research subject aggregation platform. Second, we use academic experts (graduate students or PhD holders in psychology or related disciplines) as our second data source. For our third and fourth sources, we query two frontier LLMs (GPT-4o and Claude 3 Opus). Fifth, we use a proprietary deep neural network called PersonalityMap as our last data source. We preregistered our data collection and analysis plans on the Open Science Framework (https://osf.io/g4qm9/?view_only=633604f20ba3451cbac9852a8e9e68c0). Research protocols were approved by the Institutional Review Board at New York University. Participants gave informed consent to participate in all experiments. We did not use deception.

### Data

For our test data set, we use 249 pairs of personality psychology items taken from the SAPA Personality Inventory^32^. This inventory drew on a total of “125,000 study participants from over 220 countries or regions”^32^ over the course of their exploratory, replication, and confirmatory samples. The items pairs used in our study were drawn from this larger inventory. For example, one such item pair might be “I am an extraordinary person” and “I am easily discouraged”. Overall, our data set has 103 unique items. We sampled one third of pairs to have an empirical correlation of less than −0.2, a third to have a correlation between −0.2 and 0.2, and a third to have a correlation above 0.2, in order to ensure that our test captured various types of correlations as opposed to focusing on one, e.g., small-to-nonexistent correlations between random items. For a full list of item pairs, see Supplementary Notes.

### Sample size justification

To arrive at our sample size and data numbers, we conducted the following preregistered sample size justification. Using a standard small-to-medium effect size of *f* = 0.225 as our smallest effect size of interest for the one-way ANOVA used in our aggregate-level analysis, this would require a total of 245 participants at 80% power and an alpha level of 0.05. As our aggregate-level analysis is conducted at the question level, this corresponds to a total of 245 question pairs. We use 249 question pairs to meet this target. In order to ensure at least three predictions per item for meaningful aggregation and to account for participants answering less questions than expected, we conducted a Monte Carlo simulation, where the results indicate we need to recruit at least 119 participants. To account for potential drop-out for a variety of reasons (wrong expert status, incomplete surveys, etc.) and to account for modelling uncertainty, we were aiming to recruit 250 participants in the lay participant condition to account for partial completions and to ensure that our study is well-powered. For the expert condition, we aimed to recruit 200 participants who are experts in psychological research/behavioural science (graduate students or PhD holders in these fields). Our samples were willing to answer more questions than anticipated, with the median number of responses for both conditions being 30, and at the lowest number of responses for an item pair being 16 for the lay condition and 18 for the expert condition. For the pre-registered LLM conditions, we collected three runs for each item. For PersonalityMap, we collected only a single prediction as the model is deterministic.

### Participants

We recruited a total of 254 participants via the Positly.com platform. The mean age was 46.35 years (SD = 11.83), with 56% of participants identifying as men. Participants were paid a total of $1.80 each for participation (averaging to approximately $8.40 per hour). The survey included a total of 30 randomly selected item pairs that participants were asked to evaluate the relationship between. They entered their prediction on a slider ranging from −1 to +1 in increments of 0.02. Prior to their correlation estimation task, all participants completed a short introduction to correlations that included some theoretical explanation as well as examples of large, small, and zero correlations. Participants had to correctly answer some questions about correlations (to demonstrate they understood the concepts) before proceeding, though they could try as many times as they liked until they got the correct answer. The study was implemented using the GuidedTrack.com study creation platform. The study can be viewed precisely as participants saw it here https://www.guidedtrack.com/programs/18blaeo/preview and the study code can be found and copied here: https://www.guidedtrack.com/programs/27606/edit (available after creating a free login).

Additionally, we recruited a total of 272 participants from a number of academic email lists like SJDM as well as social media platforms such as LinkedIn and X/Twitter. The mean age of our sample was 33.86 years (SD = 8.12), with 52% of participants identifying as men. Participants were academics in psychology/behavioural science, which we defined as graduate students, postdocs, or professors. Overall, 36% of participants were professors, with 20% having completed a PhD without being a professor and 44% being graduate students. All participants were presented with a total of 30 randomly selected item pairs. They entered their estimated correlations on a slider ranging from −1 to +1 in increment of 0.02. Participants were provided with the opportunity to complete the same correlation training that all lay participants had to complete, but they could straightforwardly opt out of it. Expert participants received a $5 gift card and had the option of donating the amount to charity instead. The study can be viewed precisely as expert participants saw it (https://www.guidedtrack.com/programs/18blaeo/preview?expert=1).

### PersonalityMap

We collected our correlation estimates for the specialised model from a proprietary model called PersonalityMap that was developed by the startup foundry Spark Wave. It is a supervised training model that uses a pair of psychometric personality items as input. A fully connected deep neural network tries to generate the correlation between them, and after a correlation has been produced, compares it with the target correlation, adjusting the weights according to a backpropagation-based algorithm. The network was trained for 501 epochs using 992,003 item pairs as the training data, with each assigned a numerical embedding vector. In addition, 51,336 item pairs were withheld from training and used as the test set for measuring performance. The momentum was set to 0.9, and the learning rate was linearly increased from zero to 4e-5 for one epoch, after which it was decreased using cosine for 500 epochs, at which point it became 4e-6. Weights of trainable connections were initialised using standard He initialisation. The hidden layer size was set to 5 layers in total. We collected the model’s predicted correlations on all 249 item pairs, all of which were not part of the data set used to train PersonalityMap (i.e., no data from any of the items that were part of the 249 item pairs was used to train the model).

### Large language models

We collected correlation estimates from two of the most advanced LLMs at the time of conducting this study: Claude 3 Opus (claude-3-opus-20240229) with a 200,000 token context window and training data up to August 2023 and GPT-4o (gpt-4o-2024-05-13) with a 128,000 token context window and training data up to October 2023. Each model was queried with an advanced prompt via the respective API. We queried each model at temperature *T* = 0. However, to account for the sparse mixture-of-experts architecture^33,34^ that is likely used in both models due to their non-deterministic output even at *T* = 0^35^, we queried both models three times. This allows us to partially reduce variance inherent in the batched inference approach of this architecture^36^ irrespective of the otherwise expected determinacy of *T* = 0 that would be preferable for capability assessment.

We used a zero-shot approach without in-context learning, drawing on the following prompt for runs (with the prompt for Claude 3 Opus including an additional phrase at the seventh step to ensure consistent outputs: “(Predicted Correlation: XX.XX)”). The prompt design drew heavily on current best practices of LLM prompting, including standard chain-of-thought and step-by-step reasoning^37^ to increase reasoning capabilities, while also making use of the expert-persona framing technique^38^ in order to increase model confidence and reasoning complexity. This included identification of potentially relevant psychological constructs and previous literature on these. The model was also instructed to reason from least-to-most complex justifications^39^ to consider different levels of abstraction and to take a deep breath^40^ to further improve general performance. Then, the prompt instructed the model to make use of tree-of-thoughts^41^ and to rely on a self-consistency constraint^42^ in its reasoning to consider alternative explanations, ensuring that considerations of no correlation at all are continually considered. Following this, we asked the model to think of reasons against its estimate^43^ to further induce critical reflection before proceeding to highlight the emotional stakes^44^ of this task in an attempt to increase model effortfulness, which is also what we aim to elicit with a tipping reminder and further focus on personal real-world stakes. Using GPT-4’s tokenizer, this prompt without specific items amounted to 671 tokens. For the full prompt, see Supplementary Notes.

### Reporting summary

Further information on research design is available in the Nature Portfolio Reporting Summary linked to this article.

## Results

As with our research questions, we split the results section into two parts. In the first part, we treat the machine learning model approaches as individual data points that are compared to individual lay people and academic experts. This allows us to test how well an instance of a given machine learning model approach may work compared with actual academic experts in the field or lay people. In the second part, we use simple aggregation for all conditions, using the median prediction at each question pair. This evens the playing field and allows the human approaches to draw on the wisdom of crowds of a set of heterogeneous respondents. Unless specifically indicated, all analyses below were preregistered on the Open Science Framework (https://osf.io/g4qm9/?view_only=633604f20ba3451cbac9852a8e9e68c0). For an example output of the two LLMs, see Supplementary Notes.

### Individual comparisons

To address our first research question (hypotheses 1a and 1b) of testing how well individual models do against individual humans, we randomly select one of the three LLM instances for both GPT-4o and Claude 3 Opus for both analyses. Then, we compute the absolute prediction error between the predicted correlation and the empirical correlation between the two items with lower scores indicating higher accuracy.

In Table 1, we provide descriptive results of the prediction errors, reporting means and standard deviations of average scores at the predictor level. The AI average prediction errors are calculated across all items (separately for each model run), whereas each human’s average prediction error is calculated across only the subset of items for which the human made estimates. We then average the errors for each person or model run (i.e., separate averages for each of the first, second, and third model run for the LLMs or one average for the single model run for personality map). In Table 1, we report the mean and standard deviation across these averages for each condition, though note that variability for AI models is low due to low number of runs. Note that this difference does not confer machines with a wisdom-of-crowds based advantage because we are computing the average of the errors, not the error in the average.

**Table 1:** Summary statistics of averaged absolute prediction errors

Condition	Mean	Standard deviation	Minimum	Maximum
Lay	0.29	0.09	0.11	0.65
Expert	0.20	0.09	0.08	0.86
GPT-4o	0.14	<0.01	0.14	0.15
Claude 3 Opus	0.11	<0.01	0.11	0.11
PersonalityMap	0.07	–	–	–

Mean of average scores of individual humans or model runs. For lay people and experts, we calculate the average absolute prediction errors across questions for each individual, based on the subset of questions which the relevant individual predicts. Then, treating the average prediction error for each individual as a separate observation, we calculate the mean and standard deviation across those average prediction errors. For GPT-4o and Claude 3 Opus, we calculate the average prediction error across questions separately for each of the first, second, and third runs of the relevant model, where each run includes one prediction error for every question. Then, treating the average prediction error for each of the three runs as a separate observation, we calculate the means and standard deviations across those three average prediction errors. Both LLM conditions have minimal variability due to temperature = 0 and low run count (*n* = 3). PersonalityMap is deterministic and only has a single run. Lower scores indicate higher accuracy.

We start our main analysis by testing *Null Hypothesis 1a: The machine approaches do not outperform or underperform the median percentile rank of lay humans and experts*.

For this hypothesis, we preregistered to determine the rank of machine learning model approaches in the following way. First, we take each individual’s (or model’s) predicted correlations across all item pairs and calculate the average prediction error for that individual human (or model run), leaving each participant or model run with an average error score. Note, to calculate the LLM averages, we randomly select one of the LLM’s three estimates for this analysis to avoid conferring LLMs with a wisdom-of-crowds advantage.

**Fig. 1:**
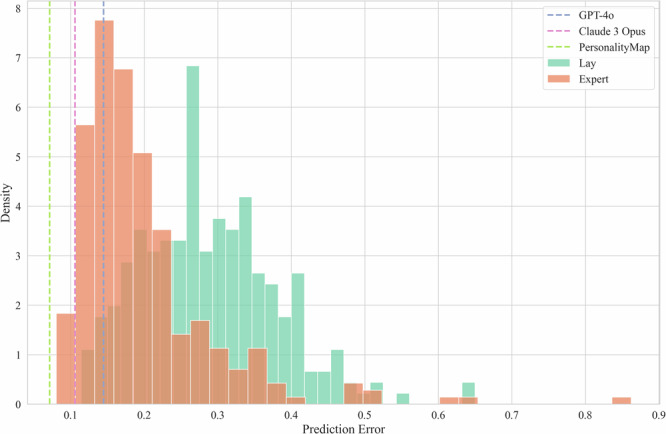
Density histogram of participant prediction errors. Note. Density histogram, with vertical lines showing the average error across all items for GPT-4o and Claude 3 Opus (because we elicited three estimates from each model for each item, we randomly select one model for all items to calculate the average) as well as for the single PersonalityMap instance, with each model showing lower error than the majority of both human populations. Human prediction errors are calculated for each individual person separately (but averaged across all that person’s predictions). 254 lay participants. 272 expert participants.

For each of the three AI approaches, we calculated the percentile rank of their average estimate with respect to both the lay and the expert human populations. Then, to test our Null Hypothesis 1a, we compute 95% confidence intervals via bootstrapping, resampling with replacement at the question level over 10,000 iterations. We find that all three AI approaches significantly outperform the preregistered 50% baseline, ranging from a rank of 70.22 (95% CI [60.66, 81.62]) for GPT-4o in comparison with the academic experts to 100 (95% CI [100, 100]) for PersonalityMap with respect to the lay population, see Table 2 for full results and Fig. 1 for a visualisation of the same analysis, showing that all AI models outperform more than half of all individual humans. This allows us to reject our first null hypothesis (1a).

**Table 2:** Percentile rank

Model	Comparison	Percentile rank CI
GPT-4o	Lay individuals	95.67 [93.31, 98.03]
	Expert individuals	70.22 [60.66, 81.62]
Claude 3 Opus	Lay individuals	100 [98.82, 100.00]
	Expert individuals	95.22 [87.50, 98.16]
PersonalityMap	Lay individuals	100 [100.00, 100.00]
	Expert individuals	100 [100.00, 100.00]

Each machine learning model approach was compared individually to each of the human populations.

**Fig. 2:**
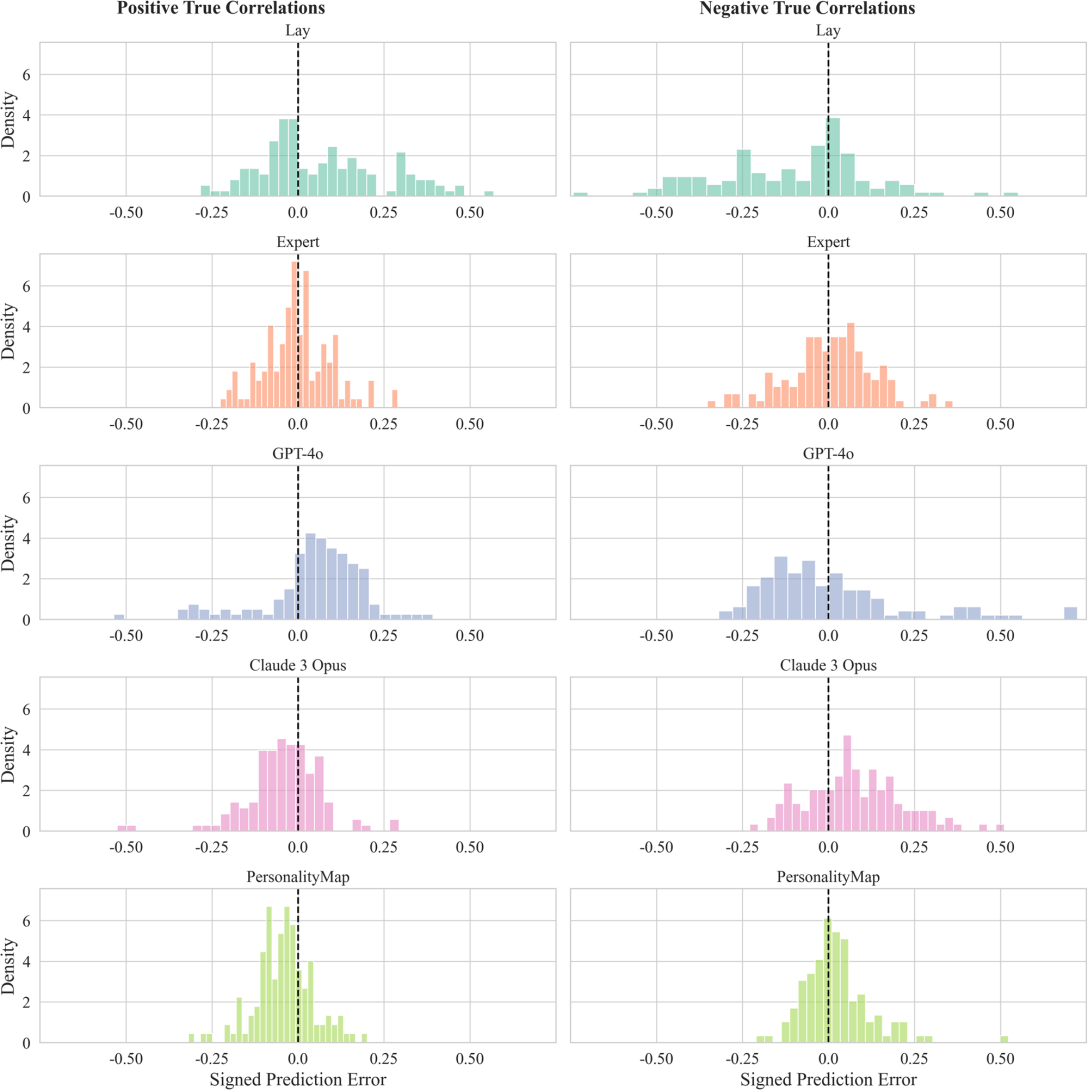
Histogram of signed prediction errors by condition and split by true correlation sign. Histogram with signed prediction errors calculated at the item level, segregated by the sign of the true correlation. The first row represents items with positive true correlations, and the second row represents items with negative true correlations. 254 lay participants. 272 expert participants.

To aid this analysis, in Fig. 2, we plot the signed prediction errors of each prediction made within each condition, now split by the sign of the true correlations (positive and negative) rather than aggregating at the person level. This approach allows us to observe the differences in the distribution of prediction errors across both positive and negative correlations. One take-away from this plot is that PersonalityMap has a heavily concentrated number of prediction errors close to 0, while all other conditions are more widely dispersed. We also find that lay participants tend to overexaggerate positive correlations and underexaggerate negative correlations, which is less present in experts, with GPT-4o showing a similar pattern and Claude 3 Opus the reverse.

Next, we test *Null Hypothesis 1b: The machine approaches do not win more or less than 50% of individual comparisons to lay humans or experts*.

To do so, we used the same randomly selected instances of GPT-4o and Claude 3 Opus, as well as the single instance of PersonalityMap as in our previous analysis We then performed an item-by-item comparison between each model and each human participant, considering only the questions answered by both the participant and the models. For each comparison, we identified common questions and recorded the prediction errors of the models and the human participants before counting the wins for each model. A “win” for a model was defined as having a lower prediction error than the human participant on more than half of the common questions, with ties resolved in favour of the human participant. In other words, there was one observation for each participant, indicating whether the given machine learning model approach outperformed the human for more than half of the estimates that the human made. We defined the win rate for each AI approach as the percentage of wins for each model, calculated separately for lay and expert populations. The results in Table 3 showed that all three models demonstrated superior performance against the lay population, each achieving a win rate of over 90%. Against the expert population, GPT-4o had a win rate of 69.85%, and PersonalityMap showed a win rate of 99.26%. All approaches are significantly different from the preregistered 50% baseline. This allows us to also reject our second null hypothesis (1b).

**Table 3:** Win rate

Model	Comparison	Win rate	Binomial test (*p*-value)
GPT-4o	Lay individuals	90.94%	<0.001
	Expert individuals	69.85%	<0.001
Claude 3 Opus	Lay individuals	97.64%	<0.001
	Expert individuals	86.40%	<0.001
PersonalityMap	Lay individuals	100.00%	<0.001
	Expert individuals	99.26%	<0.001

Binomial test is conducted against the 50% baseline, showing that all machine learning model approaches outperform this baseline and win more than half the individual matchups.

The results from the first set of analyses show that all AI approaches significantly outperform the majority of lay humans as well as academic experts. This suggests that on an individual-level comparison, LLMs as well as PersonalityMap show superior personality correlation prediction capabilities than most academic experts.

### Aggregate comparisons

Our second research question is to compare the aggregate performance of AI and human approaches based on a representative estimate for each item pair. Thus, the unit of observation is each of the 249 item pairs. We calculated variables measuring the prediction accuracy of a representative prediction from each approach for each of these item pairs. For the LLMs, for the representative prediction, we take the median of all three predictions on each item, while for PersonalityMap, we take the single prediction it provides per item. For both human conditions, we also compute the median on each item (after having removed all missing values, as each individual human participant only responded to a fraction of the total item pairs). This procedure leaves us with a single, representative prediction in each condition for each item. Then, we conduct further analysis with these predictions to answer our second research question via the following three null hypotheses.

**Fig. 3:**
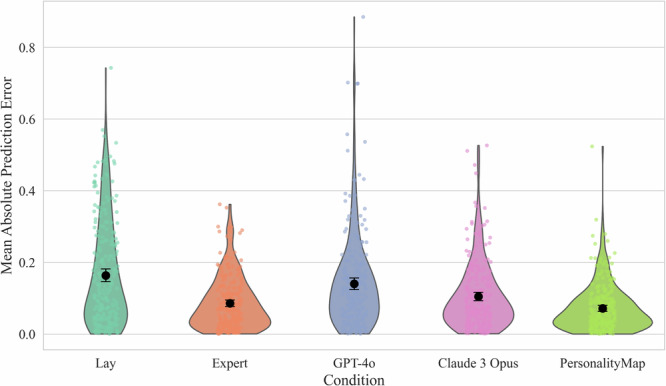
Mean Absolute Prediction Error by Condition. *Note*. Violin plots with 95% confidence intervals of mean absolute prediction error of aggregated predictions, showing that PersonalityMap and experts outperform lay people and GPT-4o. Lower bars indicate superior performance. 254 lay participants. 272 expert participants.

For *Null Hypothesis 2a: There is no difference in prediction error between the conditions*, we first tested the assumptions of normality (Shapiro–Wilk) as well as homogeneity of variances (Levene’s), showing assumption violations for both, *p* < 0.001. Based on these results, we conducted a Kruskal–Wallis *H*-test, finding significant differences between the conditions. H(4) = 90.84, *p* < 0.001. This allows us to reject Null Hypothesis 2a. See Fig. 3 for a visualisation of the mean prediction error for each condition.

We then ran a Dunn’s post-hoc test, employing a Bonferroni correction for multiple comparisons. We find that Claude 3 Opus outperforms both GPT-4o, *p* = 0.011, and the lay population, *p* < 0.001, but is outperformed by PersonalityMap, *p* < 0.001, with no significant differences to the academic expert sample, *p* = 0.329. PersonalityMap is similarly not significantly different from the expert population, *p* = .444, but outperforms all other conditions at medium-to-large effect sizes with Hedge’s g between 0.41 and 0.82. GPT-4o is not statistically different from the lay population, *p* = 1.000, significantly underperforming all other conditions at medium effect sizes with Hedge’s between 0.31 and 0.66. See Table 4 for the full pairwise comparison results with adjusted p-values.

**Table 4:** Post-hoc pairwise comparisons

Comparison	*p*-value (adj.)	Hedge’s g
Claude 3 Opus vs Expert	0.329	0.22
Claude 3 Opus vs GPT-4o	0.011	−0.31
Claude 3 Opus vs Lay	<0.001	−0.49
Claude 3 Opus vs PersonalityMap	<0.001	0.41
Expert vs GPT-4o	<0.001	−0.51
Expert vs Lay	<0.001	−0.68
Expert vs PersonalityMap	0.444	0.20
GPT-4o vs Lay	1.000	−0.17
GPT-4o vs PersonalityMap	<0.001	0.66
Lay vs PersonalityMap	<0.001	0.82

The *p*-values are adjusted via the Bonferroni correction.

For *Null Hypothesis 2b: There is no difference in prediction correlation between the conditions*, we test whether the conditions differ in their correlation between the aggregate predictions and the empirical correlation values. To better allow for comparisons between correlations that might all be at the upper end of the range, we normalise the correlations via the Fisher’s *Z* transformation prior to computing 95% confidence intervals via bootstrapping with 10,000 iterations, where we resample the questions. We can reject Null Hypothesis 2b as we find differences between some conditions. For correlation coefficients and Fisher’s *Z* values with bootstrapped 95% confidence intervals, see Table 5.

**Table 5:** Pearson correlations and Fisher’s *Z* values

Condition	Pearson correlation	Fisher’s *Z* (95% CI)
Lay	0.88	1.37 (1.25, 1.50)
Expert	0.90	1.45 (1.33, 1.57)
GPT-4o	0.78	1.05 (0.87, 1.25)
Claude 3 Opus	0.80	1.11 (0.96, 1.26)
PersonalityMap	0.91	1.52 (1.35, 1.69)

Bootstrapping resamples the questions, showing that PersonalityMap and Expert correlations are higher than the other approaches’.

We can get a sense of which approaches statistically outperform other approaches based on whether there is overlap in their Fisher’s *Z* 95% confidence intervals in Table 5. These confidence intervals indicate that, in terms of the relationship between predicted correlations and actual empirical correlations, PersonalityMap outperforms both GPT-4o and Claude 3 Opus, with both human conditions being not statistically different from it. There is also evidence of human samples showing higher correlations than both GPT-4o and Claude 3 Opus, although there is some very slight overlap between the lay population’s confidence interval and those of each LLM.

**Fig. 4:**
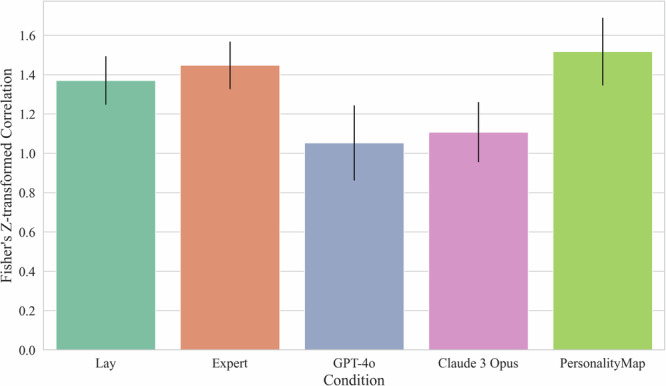
Prediction correlation by condition. Plot shows Fisher’s *Z*-transformed correlations and bootstrapped 95% confidence intervals of aggregated predictions between predicted and empirical correlations, showing that LLM predictions are less correlated with empirical correlations than the other conditions. 254 lay participants. 272 expert participants.

It is notable that this analysis is the first in which lay predictors appear to outperform LLMs. This outperformance arises in part because of the aforementioned aggregation effects, as non-aggregated correlations for the human samples would be much lower, at 0.68 and 0.70 for the lay and expert populations respectively. However, this aggregation is unlikely to be the whole explanation, as even in our analyses of aggregated prediction errors, LLMs (particularly Claude 3 Opus) appeared to outperform humans. Why might LLMs have lower average prediction errors yet also have estimates less correlated with the truth? We speculate that machine learning model approaches are well calibrated to the magnitude of personality-item correlations in general, and thus make estimates of a similar range of magnitudes to that of the correlations we observe. This advantage reduces average errors compared to humans (e.g., a human who predicted a correlation of 0.99 for a true correlation of 0.1 and −0.99 for a correlation of −0.1 one would have very bad average errors compared to a machine learning model that estimated, say, 0 and −0.15 respectively). In contrast, humans might be better at anticipating the relative magnitude and direction of different types of relationships. This human advantage improves the correlation between their predictions and the truth (e.g., a human who predicted a correlation of 0.99 for a true correlation of 0.1, 0 for a true correlation of 0, and −0.99 for a correlation of −0.1 would have a superior correlation with the truth than an AI that gave the more-accurate-but-incorrectly-ordered predictions of 0, 0.15, and −0.15 respectively). Interestingly, while experts vastly outperform lay people in terms of average prediction error, there is no detectable difference between experts and lay predictors in terms of the correlation of their predictions with the truth. This distinction hints that experts’ advantage over lay people is more driven by appreciation of the typical magnitude of personality-item correlations rather than insight into the direction or strength of relationships. See Fig. 4 for a graphical overview of these results.

**Fig. 5:**
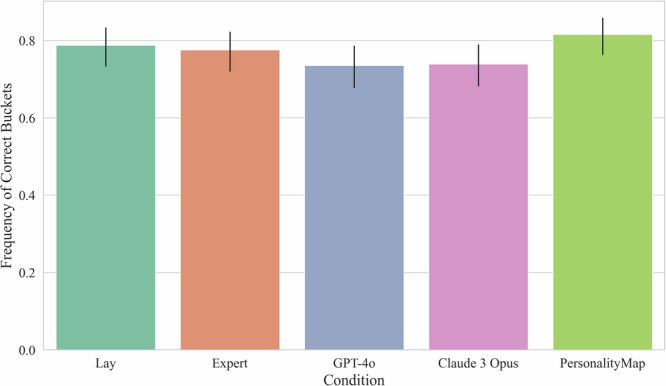
Frequency of correct buckets by condition. Bar plot with 95% confidence intervals of aggregated predictions, showing no differences in correct frequency across conditions. 254 lay participants. 272 expert participants.

For our analysis of *Null Hypothesis 2c: There is no difference in bucketised prediction error between the conditions*, we analyse the frequency with which the different approaches’ predictions fall into the correct bucket as opposed to using prediction error with the detailed empirical correlation. For this, we classified predictions as falling or not falling into the following buckets: [<−0.1; −0.1 to 0.1; >0.1]. We then conduct a Chi-square test of independence, where we fail to find statistically significant differences between the conditions, *χ*² (4, *N* = 1245) = 6.42, *p* = 0.170, see Fig. 5. As such, we are unable to reject our Null Hypothesis 2c.

This result suggests that much of the prediction advantage that a system like PersonalityMap has over human predictors lies in the ability to determine the size of correlations, rather than simply whether they are positive, negative, or close to zero. While in some situations, only the direction is important, in other situations it’s useful to be able to distinguish between small correlations, moderate correlations, and large correlations, such as when using items to make predictions, and such as when constructing scales. Additionally, even in situations where only a directional prediction is useful, where AI predictors may not be substantially more accurate than experts, AI systems have a dramatic speed and cost advantage. They are especially more time and cost efficient than taking the median prediction of experts, which in many cases is unrealistic to obtain.

### Additional analyses

We conduct two additional sets of analysis that we conducted after the original period of data collection; we thank anonymous reviewers for these suggestions. We preregistered these new analyses. However, because we formulated these new analyses after having seen the data from our main analyses, we will treat them as exploratory (https://osf.io/g4qm9/?view_only=633604f20ba3451cbac9852a8e9e68c0). Our first set of additional analyses adds a further specialised machine learning model, SurveyBot3000^20^, to the comparisons to also test different specialised models and compare them to the results presented in our preregistered analyses. Second, to further investigate the potential of using several runs of LLMs, we also add GPT-4o with a higher temperature setting (aggregating them via the median to see if that outperforms a temperature of zero).

SurveyBot3000^20^ is a fine-tuned model that uses the sentence transformer all-mpnet-base-v2 as the pre-trained model, that was then fine-tuned in two stages: polarity calibration and domain adaptation. Their pilot results showed strong accuracy in predicting empirical inter-item correlations, as well as scale reliabilities and inter-scale correlations. We tested SurveyBot3000’s performance also on our item pairs to further contextualise the results of PersonalityMap and our set of LLMs. We find that SurveyBot3000 exceeds the performance of human experts in individual comparisons and is not statistically different from them in aggregate analyses. While PersonalityMap’s performance point estimates are superior to those of SurveyBot3000 on three of our performance measures (correlation, mean error, and win rate against experts), the differences are small and not statistically significant - making it appear that SurveyBot3000 is on par with PersonalityMap. However, the interpretation of these findings is very tentative since SurveyBot3000, in contrast to PersonalityMap, was trained on the SAPA^20^. This means that our test set was part of SurveyBot3000’s training data, making direct comparisons to our models not possible since the reported performance here is compromised by data contamination (i.e., we are unable to tell if SurveyBot3000’s performance is due to genuine generalisation ability, or simple memorization, since it was trained on the answers that we use to test the performance of all our methods). For completeness, we report SurveyBot3000’s performance on our preregistered hypotheses in Supplementary Methods.

Second, we also ran an additional condition with the same GPT-4o model that we preregistered (gpt-4o-2024-05-13), but at a high temperature (of 1) with 30 runs for each item (which we then take the median of), to further test current frontier model performance at different parameters. Temperature is a hyperparameter for LLMs that controls the randomness of the model’s predictions—at higher temperatures models are more “creative”, producing more unique and unusual responses. While higher temperatures typically produce worse performance, one strategy to try to improve LLM performance is to generate more responses at higher temperature and then combine them (e.g., through taking the median). Of course, this also comes at greatly increased cost, since the LLM must be run many times. We find that for most of our preregistered comparisons, the high-temperature version of GPT-4o with 30 runs is on par with the low-temperature version at 3 runs. However, we find that in the aggregate analysis, drawing on the more varied predictions from higher temperature leads to improved performance on one of our measures. Specifically, with respect to the correlation between median predicted correlation (across the different runs of the same model) and the empirical correlation, the high-temperature multiple-runs condition of GPT-4o is not statistically different from PersonalityMap, whereas the low-temperature version of GPT-4o was statistically worse. For a full set of results of this model on our preregistered hypotheses, see Supplementary Methods.

The addition of the high-temperature version of GPT-4o also allows us to test the interrater reliability of our LLMs, specifically between the low-temperature GPT-4o queries at three runs per item and the high-temperature GPT-4o queries at 30 runs per item. In exploratory analyses, we calculated the Intraclass Correlation Coefficient (ICC) using a two-way random-effects model to assess the consistency of the models’ predictions across multiple runs. The ICC(2,1), representing the reliability of a single run, was higher for the low-temperature GPT-4o (ICC(2,1) = 0.874) compared to the high-temperature GPT-4o (ICC(2,1) = 0.826). This indicates that individual runs at low temperature produce more consistent predictions than individual runs at high temperature, as expected due to the increased randomness introduced by higher temperature settings. However, when considering the reliability of the average predictions across runs, the ICC(2,k) shows a different picture. The ICC(2,k) for the low-temperature GPT-4o (with *k* = 3 runs) was 0.954, while the ICC(2,k) for the high-temperature GPT-4o (with *k* = 30 runs) was 0.993. For comparison, Claude 3 Opus had an ICC(2,k) value of 0.987. Despite the higher variability in individual runs at high temperature, the larger number of runs significantly enhances the reliability of the average prediction. This occurs because averaging over more runs reduces the impact of random errors, leading to a more stable and consistent aggregate prediction. Therefore, while high-temperature settings introduce more variability in single outputs, the aggregation of multiple runs yields highly reliable results, even surpassing the reliability of fewer low-temperature runs.

## Discussion

Our results suggest that current AI models are roughly as good as, if not better, than human experts in predicting correlations amongst human personality questionnaire items. Specifically, we found that AI models perform much better than the vast majority of individual lay people as well as academic experts. However, expert groups can match specialised AI model performance when their predictions are aggregated by taking the median prediction for each question, and are able to exceed LLM performance. This effect showcases the wisdom of crowds of human populations, i.e., the phenomenon where the collective judgement of a group is often more accurate than that of individual members, which is a classic finding in forecasting studies generally^45^. In simple terms, current frontier LLM performance (with respect to predicting personality associations) is currently somewhere between what an individual person can do and what a group of experts can do together at the specific tasks we studied. Crucially, we also found that a specialised AI system trained on personality data (i.e., PersonalityMap) performed better than generalised AI systems (i.e., current frontier LLMs), outperforming even aggregate expert estimates.

By comparing LLMs and PersonalityMap to the full set of individual lay people and academic experts, we find that all machine learning model approaches are significantly better than the median human counterpart and win significantly more than 50% of 1-on-1 comparisons. In fact, PersonalityMap and Claude 3 Opus outperform at least 85% on both of these metrics. This suggests that when individual models are employed in a task like this, one can expect them to outperform most individual human experts that one may have consulted otherwise. This result is consistent with findings from Peters and Matz^23^, who found that LLMs could accurately infer Big Five personality traits from social media data or free-form user interactions^46^, suggesting that these models are adept at interpreting human psychological indicators across a number of contexts. Our evidence thus fits with previous work and reinforces the broader trend of machine learning models becoming adept at human personality tasks, sometimes reaching human expert performance. These findings might open up potential applications of machine approaches, both for general LLMs but especially for targeted ones like PersonalityMap and SurveyBot3000, with the former also being able to facilitate a back-and-forth between the human and the model via the now well-known chat bot settings in which LLMs are often encountered, see Supplementary Notes, and with the latter able to provide accurate predictions that may help expert applications.

Contrary to this rather clean result, the data collected to answer our second set of research questions, focusing on aggregate predictions, is considerably more mixed. For these analyses, we use one representative prediction within each condition, i.e., each condition has the median prediction per item pair. Testing for the differences between the average accuracy of these aggregated forecasts, we find that GPT-4o is not statistically different from the set of lay individuals, while Claude 3 Opus and PersonalityMap are better than both but remain not statistically difference from the academic expert sample. This pattern is somewhat replicated with respect to the outcome measure of correlations between predicted and empirical correlation values, where PersonalityMap is not statistically different from the human approaches, all outperforming the two LLMs. On our last measure of bucketised accuracy, we fail to find any differences, suggesting that what makes the best prediction methods stand out is not whether they get the direction of predictions correct, but how close they are to the actual correlation values.

One question that arises is why do the LLMs appear to have a reduced advantage in the aggregated set of research questions? We speculate that the main reason for these different patterns of results is that in the second set of analyses, the human estimates are boosted by the fact that they can rely on the wisdom of the crowd^47^, a well-established phenomenon that shows that aggregate values can cancel out individual errors and improve predictions across a wide set of contexts. Specifically, for humans, we take the median of the humans’ estimates, which would otherwise be subject to much more noise. The machine estimates are subject to less noise (zero in the case of Personality Map) and so taking the median estimate does less to improve accuracy as the aggregation occurs only over three judgments for the LLMs that were already produced with a temperature setting at 0, compared to at least 16 judgments for the human conditions, with median aggregation being over at least 30 judgments. In related work, Trott^48^ recently developed a framework, the Number Needed to Beat which quantifies how many human participants are needed such that their aggregation matches LLM performance. The main take-away from our results is that, in contrast to individual comparisons, as an aggregate, academic experts are not outperformed by the machine learning model approaches at the current margin. However, acquiring the necessary results from a human academic expert sample is expensive and time-consuming, while many machine learning model approaches are much cheaper and easier to implement. Strong AI performance when compared to individual humans but underperformance or indistinguishability has also been observed in the context of geopolitical and economic forecasting^49^.

Although PersonalityMap performed very well on all measures, it is also notable that humans (and particularly lay people) performed relatively better vs. LLMs in terms of correlation and bucketised predictions than in terms of average prediction error. We speculate that this difference arises from the different strengths and weaknesses of the various approaches. Lay people appear to be particularly poorly calibrated as to the magnitude of correlations generally, but (when their judgments are aggregated) better than LLMs and close to experts in terms of their judgments of the relative strength and direction of specific correlations. In contrast, LLMs appear weaker than (aggregated) experts and lay people in terms of their judgments of the relative strength and direction of specific correlations, but superior to both human populations in their calibration to the typical magnitudes of correlations generally in our sample. These differences in strengths and weaknesses are somewhat intuitive and in line with the view that AI applications face a jagged landscape, with some specific tasks and comparisons favouring AI and others disfavouring AI^50^. We might expect lay people to have very good instincts for which kind of items might draw similar responses from a human responder. Yet, we might expect LLMs, and to some extent experts, to have been exposed to far more examples of similar correlations, lending them a considerable advantage in judging their magnitude. Future research could explore these conjectures more, and perhaps test how much human judgments can be improved by exposure to examples of similar correlations.

One possibility for why AI systems may exceed human performance so much more in terms of absolute error than when predicting the correct buckets could be that AI systems model not just true underlying correlations between traits but also method factors or design-specific biases that can be associated with the use of self-reported responses—and these biases are much more impactful in terms of absolute error than in terms of predicting the correct bucket. On the other hand, if we interpret the prediction task as predicting what the actual measured correlation will be between items, then such biases are precisely part of what is intended to be predicted. While it is sometimes advantageous to calculate correlations between, for example, a self-reported personality question and an objectively measured item (e.g., income from pay stubs) to help reduce some biases, calculating correlations between self-reported items is still a commonly used procedure for a wide variety of purposes, and any such biases that exist in self-reporting will be captured in those correlations as well.

AI systems like PersonalityMap, GPT-4o, or open source LLMs^51^, which can outperform even human experts on some measures of predicting psychological facts about humans, might open an intriguing possibility for the future of social science research. To make an analogy, imagine if biologists could only ever conduct research in human bodies (in vivo) without the ability to do experiments in vitro. In such cases, research in biology would be slowed tremendously. Test tube experiments allow for much faster iteration than is possible with direct human experiments (though of course, preliminary test tube results must ultimately be confirmed in humans). But no such in vitro approach to psychology experimentation has existed, until now. Systems like PersonalityMap and other AI based predictors might be able to act as a “digital test tube” where research can be rapidly performed and iterated. With such technologies, it may be possible for researchers to generate new hypotheses and conduct preliminary tests of hypotheses before conducting a single human experiment, which may accelerate the speed of research. For example, Manning, Zhu, & Horton^52^ used LLMs in combination with structural causal models to automate the generation, simulation, and testing of social science hypotheses by structuring experiments in scenarios like negotiations and auctions, where the system predicted causal relationships and tested them through in silico simulations. Of course, as with biological experiments in test tubes, before the research is finished, the findings must be confirmed in real humans to make sure they apply. Still, if early pilot studies could be replaced with queries to machine learning algorithms, it seems possible that the research process would be accelerated.

On the other hand, there are still many limitations of AI systems for personality research. While the website hosting PersonalityMap (https://personalitymap.io/) allows researchers to see predictions about the correlations between agreement to any pair of statements, as well as the ability to control for variables and conduct simulated linear regression between an independent variable and multiple dependent variables, much of what social science is interested in is causal relationships, and this technology only allows associations to be studied. Additionally, while such a system can, in theory, already make predictions about any statements whatsoever, the accuracy of such a specialised system is likely to suffer dramatically for statements that are very different from any seen during training (a fundamental challenge for machine learning models generally). Future research will also be needed to quantify the uncertainty in these predictions so that models can make researchers aware of when the predictions are more reliable and when they are less trustworthy. Finally, current incarnations of this technology only include linear relationships (represented by correlations). Perhaps future versions will also enable the modelling of non-linear relationships as well.

### Limitations

One potential limitation of our study is that the data used, the SAPA Personality Inventory^32^, may be part of the training data for both LLMs, GPT-4o and Claude 3 Opus. While we can be sure that this is not the case for PersonalityMap, as the studied item pairs were not part of the training data, it is possible that they have been part of the LLM training data which may thus overstate their ability to predict correlations between the sets of personality items. However, we want to point out that we were not able to find these individual correlations in the public domain, making it at least relatively plausible that they were not part of the training data. Additionally, for model results like SurveyBot3000, this limitation is more severe as it was trained on what ended up being our test set.

Another limitation is that our results are also limited by the choice of data we use to test the models and humans on, reducing generalisability. Specifically, we only use a single source to draw our item pairs from (the SAPA Inventory), resulting in a single item type of self-descriptive statements based on self-report in only one mode of assessment (cross-sectional). A further constraint on the generalisability of our findings is that we drew on a W.E.I.R.D. sample^53,54^. These concerns limit how much we can generalise from our results. It is plausible that different items might lead to different results that may not replicate in different samples. However, as self-report items remain the backbone of contemporary personality psychology, we believe that, at least with respect to the current paradigm, our results should hold up.

A third limitation is that we did not incentivise either of our human condition’s responses for accuracy. This may reduce their performance, which is something that future research can test.

Fourth, given that prediction questions were randomly assigned to humans, some correlations would be displayed to humans more than to machines and some less. While such differences would reduce the precision of our estimates of relative difference between human and AI approaches, since they would be random, this added noise is accounted for in our confidence intervals and significance tests

### Practical implications

The results of this study highlight several potential practical implications for both psychometric research and applied fields such as human resources, healthcare, and marketing. Specialised AI models like PersonalityMap might be able to greatly expedite the research process by reliably predicting personality trait correlations, facilitating faster hypothesis testing and scale development at a lower cost. AI might also be able to also assist in automating personality assessments, reducing the need for expert input in contexts such as hiring and diagnostics. However, while generalised LLMs offer broad applicability, they still seem less effective than domain-specific models in tasks requiring high accuracy.

## Conclusion

The data presented in this paper show that generalised AI models like PersonalityMap outperform frontier LLMs as well as match human expert capabilities in predicting personality trait correlations. These results suggest that significant gains in psychometric research and practical applications may be opened up when AI applications are used in personality research and applications.

## Supplementary information

Transparent Peer Review file

Supplementary Information

Reporting Summary

## Data Availability

The data needed to replicate the analyses for this study is available in the Open Science Framework (OSF) repository at https://osf.io/kzgy7/files/osfstorage. The study interface can be seen here: https://www.guidedtrack.com/programs/18blaeo/preview, with the study code being available here: https://www.guidedtrack.com/programs/27606/edit.

## Code availability

The code needed to replicate the analyses for this study is available in the Open Science Framework (OSF) repository at https://osf.io/kzgy7/files/osfstorage.
