# Supplementary material for: AI can outperform humans in predicting correlations between personality items
Source: Commun Psychol. 2025 Feb 12;3:23. doi: 10.1038/s44271-025-00205-w (PMC11822111; doi:10.1038/s44271-025-00205-w)
Supplement: Supplementary file 2 — Supplementary Information [file 44271_2025_205_MOESM2_ESM.pdf]

Supplementary Information

Supplementary Notes

Supplementary Table 1

Full Item List

Pair	Item 1	Item 2
1	I am interested in people	When with a group, I have difficulties selecting a good topic to talk about
2	I bottle up my feelings	I don't finish the things that I start
3	I do things by the book	I don't worry about political and social problems
4	I make a fool of myself	I am always ready to start afresh
5	I like to do frightening things	I am sensitive to the needs of others
6	I don't know why I do some of the things I do	I speak softly
7	I make rash decisions	I see that rules are observed
8	I believe that others have good intentions	I feel healthy and vibrant most of the time
9	I don't like the idea of change	I do not enjoy going to art museums
10	I like to be thought of as a normal kind of person	I do things by the book
11	I love luxury	I put on a show to impress people
12	I know how to captivate people	I am exacting in my work
13	I dislike people who don't know how to behave themselves	I try to avoid complex people
14	I try to avoid complex people	When with a group, I have difficulties selecting a good topic to talk about
15	I will not probe deeply into a subject	I don't worry about things that have already happened
16	I am always ready to start afresh	I make people feel at ease
17	I would like for other people to be afraid of me	I am an extraordinary person
18	I can't stand weak people	I am a creature of habit
19	I am not good at deceiving other people	I can't stand weak people
20	I do things in a half-way manner	I make a fool of myself
21	I believe that I am better than others	I am sensitive to the needs of others
22	I have some bad habits	I carry out my plans
23	I like to get lost in thought	I need a creative outlet
24	I would like for other people to be afraid of me	I am a talkative person
25	I am easily discouraged	I stumble over my words
26	I am considered to be kind of eccentric	I put on a show to impress people
27	I am afraid to draw attention to myself	I laugh a lot
28	I seek to influence others	I love a good fight
29	I do too little work	I feel that others misunderstand me
30	I am not good at deceiving other people	I am considered to be kind of eccentric
31	I make rash decisions	I seldom get mad
32	I warm up quickly to others	I stumble over my words
33	I don't finish the things that I start	I carry out my plans
34	I often feel uncomfortable around others	I feel healthy and vibrant most of the time
35	I don't know why I do some of the things I do	I like to take it easy
36	I do things by the book	I do things according to a plan
37	I don't know why I do some of the things I do	I can be stirred up easily
38	I enjoy feeling 'close to the earth'	I am always ready to start afresh
39	I am a talkative person	I experience my emotions intensely
40	I jump into things without thinking	I feel that others misunderstand me
41	I am easily discouraged	I don't worry about things that have already happened
42	I like to get lost in thought	I love to eat

43	I enjoy interactions less than others	I am easily discouraged
44	I am easily distracted	I stick to my chosen path
45	I don't finish the things that I start	I am easily discouraged
46	I stick to my chosen path	I am mainly interested in money
47	I am interested in people	I am a very private person
48	I enjoy interactions less than others	I work hard
49	I am interested in people	I often feel uncomfortable around others
50	I like to do frightening things	I feel healthy and vibrant most of the time
51	I keep my cool	I do things without thinking of the consequences
52	I do things in a half-way manner	I do things according to a plan
53	I amuse my friends	I am sensitive to the needs of others
54	I like to be thought of as a normal kind of person	I act without thinking
55	I believe that I am better than others	I do things by the book
56	I am skilled in handling social situations	I don't finish the things that I start
57	I am skilled in handling social situations	I carry out my plans
58	I am interested in people	I rarely get caught up in the excitement
59	I am an extraordinary person	I am a very private person
60	I keep my cool	I can be stirred up easily
61	I love luxury	I only feel comfortable with friends
62	I make rash decisions	I am exacting in my work
63	I am considered to be kind of eccentric	I stick to the rules
64	I seek to influence others	I take control of things
65	I see that rules are observed	I seldom get mad
66	I seek to influence others	I rebel against authority
67	I believe that others have good intentions	I carry out my plans
68	I like to get lost in thought	I feel others' emotions
69	I believe that others have good intentions	I enjoy interactions less than others
70	I bottle up my feelings	I stick to the rules
71	I don't finish the things that I start	I feel that others misunderstand me
72	I am easily discouraged	I laugh a lot
73	I don't like the idea of change	I enjoy interactions less than others
74	I am often worried by things that I said or did	I don't worry about things that have already happened
75	I am exacting in my work	I do too little work
76	I only feel comfortable with friends	I stumble over my words
77	I feel that others misunderstand me	I have often gone against my parents' wishes
78	I am skilled in handling social situations	I laugh a lot
79	I suspect facial expressions show when sad	I don't know why I do some of the things I do
80	I am a goal-oriented person	I make people feel at ease
81	I like to take it easy	I work hard
82	I know how to captivate people	I often feel uncomfortable around others
83	I am a talkative person	I only feel comfortable with friends
84	I reveal little about myself	I can't stand weak people
85	I am not interested in abstract ideas	I speak softly
86	I enjoy interactions less than others	I am considered to be kind of eccentric
87	I bottle up my feelings	I speak softly
88	I warm up quickly to others	I feel that others misunderstand me
89	I do things in a half-way manner	I am a goal-oriented person
90	I would like for other people to be afraid of me	I like to do frightening things
91	I often feel uncomfortable around others	I don't worry about things that have already happened
92	I feel that others misunderstand me	I am afraid to draw attention to myself
93	I would like for other people to be afraid of me	When with a group, I have difficulties selecting a good topic to talk about
94	I am more easy-going about right and wrong than most people	I am a very private person
95	I am a goal-oriented person	I do too little work

96	I make rash decisions	I am easily discouraged
97	I am more easy-going about right and wrong than most people	I stick to the rules
98	I dislike people who don't know how to behave themselves	I am mainly interested in money
99	I don't know why I do some of the things I do	I don't worry about things that have already happened
100	I keep my cool	I only feel comfortable with friends
101	I know how to captivate people	I am easily discouraged
102	I am skilled in handling social situations	I don't know why I do some of the things I do
103	I feel that I have a lot of inner strength	I often feel uncomfortable around others
104	I make a fool of myself	I feel that others misunderstand me
105	I am a talkative person	When with a group, I have difficulties selecting a good topic to talk about
106	I take control of things	I rarely get caught up in the excitement
107	I make rash decisions	I am afraid to draw attention to myself
108	I do things in a half-way manner	I often feel uncomfortable around others
109	I am a goal-oriented person	I see that rules are observed
110	I seldom get mad	I feel healthy and vibrant most of the time
111	I listen to my conscience	I do things without thinking of the consequences
112	I feel that others misunderstand me	I take control of things
113	I believe that others have good intentions	I rarely get caught up in the excitement
114	I don't know why I do some of the things I do	I feel that I have a lot of inner strength
115	I dislike people who don't know how to behave themselves	I enjoy interactions less than others
116	I am skilled in handling social situations	I have some bad habits
117	I believe that others have good intentions	I don't like the idea of change
118	I can be stirred up easily	I don't worry about things that have already happened
119	I know how to captivate people	I only feel comfortable with friends
120	I like to get lost in thought	I tend to vote for liberal political candidates
121	I jump into things without thinking	I act without thinking
122	I suspect facial expressions show when sad	I jump into things without thinking
123	I like to do frightening things	I work hard
124	I am a talkative person	I feel that I have a lot of inner strength
125	I am a talkative person	I love a good fight
126	I don't know why I do some of the things I do	I have some bad habits
127	I can't stand weak people	I don't worry about things that have already happened
128	I do things in a half-way manner	I feel healthy and vibrant most of the time
129	I see beauty in things that others might not notice	I do things without thinking of the consequences
130	I break my promises	I do things without thinking of the consequences
131	I like to get lost in thought	I do things by the book
132	I laugh a lot	I feel healthy and vibrant most of the time
133	I would like for other people to be afraid of me	I rebel against authority
134	I make rash decisions	I like to do frightening things
135	I make rash decisions	I act without thinking
136	I am not bothered by disorder	I need a creative outlet
137	I do not enjoy going to art museums	I feel others' emotions
138	I break my promises	I keep my cool
139	I do things in a half-way manner	I take control of things
140	I am always ready to start afresh	I like to take it easy
141	I will not probe deeply into a subject	How often do you exercise?
142	I would like for other people to be afraid of me	People have said that I sometimes act rashly
143	I like to get lost in thought	I will not probe deeply into a subject
144	I break my promises	I work hard
145	I love a good fight	When with a group, I have difficulties selecting a good topic to talk about
146	I seek to influence others	I enjoy interactions less than others

147	I break my promises	I feel that I have a lot of inner strength
148	I see beauty in things that others might not notice	I demand quality
149	I see that rules are observed	I carry out my plans
150	I reveal little about myself	I am a very private person
151	I do things in a half-way manner	I am not easily disturbed by events
152	I feel that I have a lot of inner strength	I make people feel at ease
153	I am skilled in handling social situations	I work hard
154	I don't know why I do some of the things I do	I listen to my conscience
155	I do not enjoy going to art museums	I need a creative outlet
156	I boast about my virtues	I feel others' emotions
157	I often feel uncomfortable around others	I rarely get caught up in the excitement
158	I believe that others have good intentions	I am a talkative person
159	I am easily distracted	I stumble over my words
160	I act without thinking	I put on a show to impress people
161	I suspect facial expressions show when sad	I do too little work
162	I make rash decisions	I do things according to a plan
163	I don't like the idea of change	Are you in a committed relationship?
164	I like to do frightening things	I have often gone against my parents' wishes
165	I am a talkative person	I enjoy interactions less than others
166	I keep my cool	I put on a show to impress people
167	I seek danger	How often do you exercise?
168	I rebel against authority	I am afraid to draw attention to myself
169	I don't know why I do some of the things I do	I carry out my plans
170	I try to avoid complex people	I often feel uncomfortable around others
171	I feel that I have a lot of inner strength	I act without thinking
172	I amuse my friends	I act without thinking
173	I know how to captivate people	I am more easy-going about right and wrong than most people
174	I rebel against authority	I do things without thinking of the consequences
175	I know how to captivate people	I believe that others have good intentions
176	I am exacting in my work	I see that rules are observed
177	I warm up quickly to others	I don't consider myself religious
178	I listen to my conscience	I feel that I have a lot of inner strength
179	I make a fool of myself	I can be stirred up easily
180	I do things in a half-way manner	I am exacting in my work
181	I jump into things without thinking	I rarely get caught up in the excitement
182	I suspect facial expressions show when sad	I am considered to be kind of eccentric
183	I carry out my plans	I feel that I have a lot of inner strength
184	I make rash decisions	I feel healthy and vibrant most of the time
185	I only feel comfortable with friends	I speak softly
186	I am interested in people	I cry during movies
187	I am afraid to draw attention to myself	I am easily discouraged
188	I am not interested in abstract ideas	I warm up quickly to others
189	I don't like the idea of change	I am often worried by things that I said or did
190	I would like for other people to be afraid of me	I believe that I am better than others
191	I would like for other people to be afraid of me	I am not good at deceiving other people
192	How often do you smoke?	I am an extraordinary person
193	I keep my cool	I make people feel at ease
194	I make a fool of myself	I make rash decisions
195	I am a talkative person	I rarely get caught up in the excitement
196	I am considered to be kind of eccentric	I have often gone against my parents' wishes
197	I make rash decisions	I carry out my plans
198	I carry out my plans	I act without thinking

199	I make a fool of myself	I like to do frightening things
200	I reveal little about myself	I am a talkative person
201	I love luxury	I don't like the idea of change
202	I seldom get mad	People have said that I sometimes act rashly
203	I feel that I have a lot of inner strength	When with a group, I have difficulties selecting a good topic to talk about
204	I don't know why I do some of the things I do	I am easily distracted
205	I enjoy feeling 'close to the earth'	I am easily discouraged
206	I am skilled in handling social situations	I am easily discouraged
207	I don't know why I do some of the things I do	I feel that others misunderstand me
208	I can't stand weak people	I take control of things
209	I make a fool of myself	I don't know why I do some of the things I do
210	I make a fool of myself	I break my promises
211	I make a fool of myself	I like to be thought of as a normal kind of person
212	I do things in a half-way manner	I do things by the book
213	I do things in a half-way manner	I carry out my plans
214	I do too little work	I am sensitive to the needs of others
215	I reveal little about myself	I like to do frightening things
216	I seek to influence others	I boast about my virtues
217	I make rash decisions	I am sensitive to the needs of others
218	I do too little work	I like to tidy up
219	I am not interested in abstract ideas	I feel that I have a lot of inner strength
220	I would like for other people to be afraid of me	I make people feel at ease
221	I am an extraordinary person	I am easily discouraged
222	I love a good fight	I do things without thinking of the consequences
223	I can be stirred up easily	I am often worried by things that I said or did
224	I am skilled in handling social situations	I rarely get caught up in the excitement
225	I am considered to be kind of eccentric	I don't worry about things that have already happened
226	I have some bad habits	I seek danger
227	I seldom get mad	I experience my emotions intensely
228	I will not probe deeply into a subject	I feel healthy and vibrant most of the time
229	I like to get lost in thought	I like to be thought of as a normal kind of person
230	I listen to my conscience	I make people feel at ease
231	I can't stand weak people	I laugh a lot
232	I break my promises	I take control of things
233	I am not interested in abstract ideas	What is your weight in kilograms?
234	I seldom get mad	I feel that others misunderstand me
235	I seek to influence others	I put on a show to impress people
236	I like to visit new places	I do things according to a plan
237	I am passionate about causes	I am often worried by things that I said or did
238	I like to do frightening things	I do things without thinking of the consequences
239	I work hard	I stumble over my words
240	I am easily distracted	I have often gone against my parents' wishes
241	I seek danger	I have often gone against my parents' wishes
242	I am easily distracted	I am exacting in my work
243	I can't stand weak people	I tend to vote for liberal political candidates
244	I do things without thinking of the consequences	I tend to vote for liberal political candidates
245	I do things according to a plan	I experience my emotions intensely
246	I don't like the idea of change	I feel healthy and vibrant most of the time
247	I demand quality	I can be stirred up easily

248	I warm up quickly to others	I laugh a lot
249	I know how to captivate people	I love a good fight

Note. Full list of item pairs used.

GPT-4o and Claude 3 Opus prompt

You are a world-renowned expert psychologist. You have a long background in researching human psychology and are exceptional at statistical analysis, having published accurate research in the top peer-reviewed academic journals. You deeply understand how different aspects of human life and the psyche play together and are often asked to provide estimations of psychological results that you estimate correctly.

Your task is to estimate the correlation coefficient between two psychometric items. These items are sentences or parts of sentences. Their correlation can range from -1 to +1. You will provide correlation coefficients with at least two decimal points.

First, you begin by thinking step by step. Study each psychometric item in detail, examine them through the lens of the most accurate psychological theories and available data, and progressively articulate the logical steps that lead to your reasoning for potential correlations. These steps will include (but are not limited to):

a) Identifying potentially key psychological constructs within the items.

b) Drawing from empirical research that discusses the relationship between such constructs.

c) Analyzing how these constructs have been statistically correlated in past research.

d) Consider whether there is a correlation at all.

Second, you will structure your further assessment by progressing from least complex to most complex justifications. Start with fundamental psychological concepts and move up to more intricate and complex theories that require substantially more nuanced understanding.

Third, take a deep breath.

Fourth, summarise what you have learnt so far and map out different branches of reasoning paths, considering multiple potential outcomes and psychological interpretations of the correlations considered. Ensure that each branch represents a different line of thought, leading you through a decision-making process that mirrors traversing a decision tree.

Fifth, after exploring these various branches, use self-consistency to evaluate your findings. This involves reviewing the different lines of reasoning you have mapped out and identifying the most consistent outcomes across the different approaches you have investigated so far. Ensure that this cross-validation is thorough and lengthy. This should always include a detailed analysis of the size of the correlation, thinking through whether it should be large, medium, small, or non-existent, with small to non-existent correlations being the base case.

Sixth, consider three reasons against your analysis, outlining why you might be mistaken. Ensure that these reasons are fair and well-researched. Ensure that the estimates are not too extreme and generally fit within what is typically found in empirical research in this area, where many effects are small (-0.1 to 0.1).

Seventh, summarise your final estimated correlation between the two items in a step-by-step manner, building directly on your analysis above. Ensure that your final estimate is accurate and represents the most plausible estimate of a true correlation found in a human study of a United States of America (US) population (and is a single estimate, not a range, and is the only number you output). Note that this analysis is not a simple exercise but is actually extremely important to me personally and will have large impacts on my personal and professional life. We will test your answer in a real human sample, so ensure that you are correct; this matters! Recall that we are paying $20 for this!

The input will be:

Item 1: XXX

Item 2: XXX

The output will include your reasoning and the final answer:

Predicted Correlation: YYY

GPT-4o Example Output

Let's begin by analyzing the two psychometric items in detail:

Item 1: I do too little work

Item 2: I feel that others misunderstand me

Step-by-Step Analysis

Step 1: Identifying Key Psychological Constructs

Item 1: I do too little work

- This item likely taps into constructs such as:
  - **Work Ethic**: The belief in the importance of hard work and diligence.
  - **Self-Efficacy**: One's belief in their ability to succeed in specific situations.
  - **Procrastination**: The action of delaying or postponing tasks.
  - **Motivation**: The reason or reasons one has for acting or behaving in a particular way.

Item 2: I feel that others misunderstand me

- This item likely taps into constructs such as:
  - **Interpersonal Perception**: How one perceives others' understanding of them.
  - **Social Anxiety**: The fear of social situations that involve interaction with other people.
  - **Self-Esteem**: One's overall subjective emotional evaluation of their own worth.
  - **Communication Skills**: The ability to convey information to another effectively and efficiently.

Step 2: Drawing from Empirical Research

- **Work Ethic and Interpersonal Perception**: Research suggests that individuals with a strong work ethic may feel misunderstood if they perceive others as not recognizing their efforts. However, this is not a direct relationship and may be mediated by other factors such as communication skills and self-esteem.
- **Self-Efficacy and Social Anxiety**: Low self-efficacy can be associated with higher social anxiety, which in turn can lead to feelings of being misunderstood.
- **Procrastination and Self-Esteem**: Procrastination can lead to lower self-esteem, which might make individuals feel misunderstood by others.
- **Motivation and Communication Skills**: Low motivation can affect one's ability to communicate effectively, potentially leading to feelings of being misunderstood.

Step 3: Analyzing Past Research Correlations

- **Work Ethic and Social Anxiety**: Studies have shown a weak negative correlation between work ethic and social anxiety (e.g., -0.10 to -0.20).
- **Self-Efficacy and Self-Esteem**: There is a moderate positive correlation between self-efficacy and self-esteem (e.g., 0.30 to 0.50).
- **Procrastination and Self-Esteem**: There is a moderate negative correlation between procrastination and self-esteem (e.g., -0.30 to -0.40).
- **Motivation and Communication Skills**: There is a weak to moderate positive correlation between motivation and communication skills (e.g., 0.20 to 0.30).

Step 4: Considering Whether There is a Correlation

Given the constructs involved, it is plausible that there is a correlation between the two items, but it is likely to be weak to moderate. The relationship is not direct but mediated by factors such as self-esteem, social anxiety, and communication skills.

Further Assessment

Fundamental Psychological Concepts

- **Self-Esteem**: Both items can be indirectly related through self-esteem. Low self-esteem can lead to both feeling misunderstood and perceiving oneself as doing too little work.

- **Social Anxiety**: Social anxiety can lead to feelings of being misunderstood and can also affect one's work output.

More Complex Theories

- **Cognitive Dissonance**: Individuals who feel they do too little work might experience cognitive dissonance, leading them to believe that others misunderstand them to justify their behavior.

- **Attribution Theory**: People might attribute their lack of work to external factors, leading to feelings of being misunderstood.

Mapping Out Reasoning Paths

1. **Path 1**: Low self-esteem → Feelings of doing too little work → Feelings of being misunderstood.

2. **Path 2**: High social anxiety → Feelings of being misunderstood → Perception of doing too little work.

3. **Path 3**: Poor communication skills → Feelings of being misunderstood → Low motivation → Doing too little work.

Self-Consistency Evaluation

Across different lines of reasoning, the most consistent outcome is a weak to moderate correlation. The constructs involved (self-esteem, social anxiety, communication skills) suggest a relationship, but not a strong one.

Considering Reasons Against the Analysis

1. **Individual Differences**: Variability in individual experiences and personality traits might weaken the correlation.

2. **Measurement Error**: The way the items are phrased might lead to different interpretations, affecting the correlation.

3. **Contextual Factors**: External factors such as work environment and social context might influence the responses, reducing the correlation.

Final Estimated Correlation

After thorough analysis, the most plausible estimate of the correlation between the two items is:

Predicted Correlation: 0.15

This estimate reflects a weak positive correlation, consistent with the constructs and empirical research discussed.

Claude 3 Opus Example Output

Okay, let's think through this step-by-step to estimate the correlation between "I do too little work" and "I feel that others misunderstand me":

a) Key psychological constructs:

- Item 1 relates to work ethic, motivation, and productivity

- Item 2 relates to feelings of being misunderstood, social disconnection, and interpersonal relationships

b) Past research on the relationship between these constructs:

- Studies have found that social support and positive relationships at work are associated with higher job satisfaction and productivity (e.g. Viswesvaran et al., 1999)

- However, the specific link between feeling misunderstood by others and doing too little work has not been well-established empirically to my knowledge

c) Statistical correlations from past research:

- Meta-analyses show the correlation between social support and job performance is typically small to moderate, around r = 0.25 (Viswesvaran et al., 1999)

- But again, the specific variables here have not been directly correlated in published research I'm aware of

d) Considering if there is any correlation at all:

- There could plausibly be a small correlation, if feeling misunderstood leads to lower motivation and productivity at work

- However, many other factors influence work ethic and productivity beyond just interpersonal relationships

- It's also very possible there is no meaningful correlation between these two specific variables

Progressing from fundamental to more complex concepts:

- At a basic level, interpersonal relationships and work performance are separate domains

- More complex theories propose that social factors can influence work outcomes to some degree

- However, the specific subjective feeling of being misunderstood may not necessarily translate directly to objective measures of doing too little work

- Many individual and contextual factors likely moderate any relationship between these constructs

Mapping out different lines of reasoning:

- One perspective is that feeling misunderstood could reduce motivation and lead to underperformance at work, resulting in a positive correlation

- Alternatively, people could compartmentalize social and work life, resulting in no correlation

- A third view is that those who feel misunderstood may overcompensate by working harder to prove themselves, resulting in a negative correlation

- The most likely outcome based on past research is probably a small positive correlation or no meaningful correlation

Evaluating consistency and cross-validating:

- The "no meaningful correlation" outcome seems most consistent across different approaches

- Empirical research has not established a clear link between these specific variables

- Conceptually, while social factors can influence work, this specific link is tenuous

- Most effects in this area of research tend to be small to negligible

Considering reasons I might be mistaken:

- There could be certain populations or contexts where this correlation is stronger than I'm assuming

- I may be overestimating the tendency for people to compartmentalize interpersonal and work domains

- Unmeasured third variables could be causing a spurious correlation between these two items

Final estimate:

Based on a thorough analysis considering past research, psychological theories, and logical reasoning, my final estimate is that the correlation between "I do too little work" and "I feel that others misunderstand me" is likely to be small to negligible in a broad US population. Interpersonal and productivity domains are often separate, meta-analyses show social variables have only small effects on work performance, and no research has established a clear empirical link between these specific constructs. Accounting for self-consistency and potential errors in my reasoning, I predict the most plausible correlation is:

Predicted Correlation: 0.05

Supplementary Methods

SurveyBot3000 and GPT-4o (High-Temperature) Further Results and Discussion

Below, we report the performance of two additional models, SurveyBot3000 and GPT-4o at a high temperature setting of 1. We ran a single instance of SurveyBot3000 (Magnolia-psychometrics, n.d.) for all our item pairs, as the model is deterministic. For GPT-4o (gpt-4o-2024-05-13), we ran 30 unseeded queries at a default temperature level of 1 for each of the item pairs, resulting in a total of 7470 predictions with the same prompt as the preregistered LLM runs. Below we report the results of our preregistered hypotheses, with these two conditions added. We conduct the same analyses and only adjust calculations when additions of new conditions necessitate doing so, i.e., by adjusting for multiple comparisons in post-hoc tests.

First, testing Null Hypothesis 1a via the percentile rank calculations of the average estimate, comparing to both the lay and expert populations, we find that the effect of increasing temperature did not translate to better or worse performance for the randomly selected instance of GPT-4o compared to its low temperature version. SurveyBot3000 is on par with PersonalityMap, beating virtually all humans, see Appendix Table 2.

Supplementary Table 2

Percentile Rank

Model	Comparison	Percentile Rank CI
GPT-4o (Low Temperature)	Lay Individuals	95.67 [93.31, 98.03]
	Expert Individuals	70.22 [60.66, 81.62]
Claude 3 Opus	Lay Individuals	100 [98.82, 100.00]
	Expert Individuals	95.22 [87.50, 98.16]
PersonalityMap	Lay Individuals	100 [100.00, 100.00]
	Expert Individuals	100 [100.00, 100.00]
GPT-4o (High Temperature)	Lay Individuals	98.03 [94.88, 98.03]
	Expert Individuals	77.21 [65.81, 80.51]
SurveyBot3000	Lay Individuals	100 [100.00, 100.00]
	Expert Individuals	100 [99.63, 100.00]

Note. Each machine approach was compared individually to each of the human populations.

We find the same pattern of results also for Null Hypothesis 1b, which pits AI models against humans and calculates the respective win rates. Both additional conditions beat the 50%

benchmark. Qualitatively, the win rate for GPT-4o at a high temperature is at the same level as the low temperature version. Similarly, SurveyBot3000 has similar win rates to PersonalityMap, see Appendix Table 3.

Supplementary Table 3

Win Rate

Model	Comparison	Win Rate	Binomial Test (p-value)
GPT-4o (Low Temperature)	Lay Individuals	90.94%	<.001
	Expert Individuals	69.85%	<.001
Claude 3 Opus	Lay Individuals	97.64%	<.001
	Expert Individuals	86.40%	<.001
PersonalityMap	Lay Individuals	100.00%	<.001
	Expert individuals	99.26%	<0.01
GPT-4o (High Temperature)	Lay Individuals	93.70%	<.001
	Expert Individuals	71.32%	<.001
SurveyBot3000	Lay Individuals	100.00%	<0.01
	Expert Individuals	98.53	<0.01

Note. Binomial test is conducted against the 50% baseline, showing that all machine approaches outperform this baseline and win more than half the individual matchups.

Next, for our set of aggregate comparisons, we use the median prediction at each item as preregistered. For Null Hypothesis 2a, we compare the mean accuracy of these aggregate predictions, finding group differences at the Kruskal-Wallis H-test, $H(6)=124.54$, $p<0.001$, see Appendix Figure 1. Below, we outline the accuracy per condition in Appendix Table 4 and Dunn's post-hoc test with Bonferroni correction in Appendix Table 6. We find that SurveyBot3000 outperforms lay humans and LLMs, while not being statistically different from the expert humans. GPT-4o at a high temperature setting, on the other hand, does not differ from the GPT-4o model at low temperature, and fits in roughly with the LLM results of our other conditions, underperforming PersonalityMap and Experts, but beating lay humans.

Supplementary Table 4

Mean Prediction Error

Condition	Mean Prediction Error (SD)
Lay	0.16 (0.14)
Expert	0.08 (0.07)
GPT-4o (Low Temperature)	0.14 (0.13)
Claude 3 Opus	0.10 (0.09)
PersonalityMap	0.07 (0.06)
GPT-4o (High Temperature)	0.12 (0.09)
SurveyBot3000	0.08 (0.08)

Note. Mean prediction error by each condition with standard deviation in parantheses.

Supplementary Table 5

Post-Hoc Pairwise Comparisons

Comparison	p-value (adj.)	Hedge's g
Claude 3 Opus vs Expert	0.692	0.22
Claude 3 Opus vs GPT-4o (Low Temperature)	0.022	-0.31
Claude 3 Opus vs GPT-4o (High Temperature)	1.000	-0.13
Claude 3 Opus vs Lay	0.001	-0.49
Claude 3 Opus vs PersonalityMap	0.001	0.41
Claude 3 Opus vs SurveyBot3000	0.001	0.29
Expert vs GPT-4o (Low Temperature)	<.001	-0.51
Expert vs GPT-4o (High Temperature)	0.002	-0.37
Expert vs Lay	<.001	-0.68
Expert vs PersonalityMap	0.933	0.2
Expert vs SurveyBot3000	1.000	0.09
GPT-4o (Low Temperature) vs GPT-4o (High Temperature)	1.000	0.21
GPT-4o (Low Temperature) vs Lay	1.000	-0.17
GPT-4o (Low Temperature) vs PersonalityMap	<.001	0.66
GPT-4o (Low Temperature) vs SurveyBot3000	<.001	0.56
GPT-4o (High Temperature) vs Lay	0.121	-0.39
GPT-4o (High Temperature) vs PersonalityMap	<.001	0.56

GPT-4o (High Temperature) vs SurveyBot3000	<.001	0.44
Lay vs PersonalityMap	<.001	0.82
Lay vs SurveyBot3000	<.001	0.73
PersonalityMap vs SurveyBot3000	1.000	-0.09

Note. The p-values are adjusted via the Bonferroni correction.

Testing Null Hypothesis 2b, which looks at the differences in correlation between the predicted and the empirical relationships, we find the high temperature version of GPT-4o performing distinct from its low temperature runs like on previous tests. In this analysis, while the two conditions did have overlapping confidence intervals, the aggregated predictions from the high temperature variant were not statistically different from high performing models like PersonalityMap and expert humans, while low temperature GPT-4o performs worse. As before, SurveyBot3000 performs similarly at the same level, see Appendix Table 6.

Supplementary Table 6

Pearson Correlations and Fisher's Z Values

**Condition**	**Pearson Correlation**	**Fisher's Z (95% CI)**
Lay	0.88	1.37 (1.25, 1.50)
Expert	0.90	1.45 (1.33, 1.57)
GPT-4o (Low Temperature)	0.78	1.05 (0.87, 1.25)
Claude 3 Opus	0.80	1.11 (0.96, 1.26)
PersonalityMap	0.91	1.52 (1.35, 1.69)
GPT-4o (High Temperature)	0.88	1.34 (1.21, 1.51)
SurveyBot3000	0.87	1.36 (1.23, 1.52)

Note. Bootstrapping resamples the questions, showing that PersonalityMap and Expert correlations are higher than the other approaches'.

Lastly, with respect to Null Hypothesis 2c, testing the differences in frequency of predicted relationships in the right buckets, we continue to fail to find group differences, χ^2 (6, N = 1743) = 6.42, p = 0.377.

The results drawn from these additional analyses show two things: First, higher temperature variants of the same LLM sometimes fails to improve upon the performance of low temperature queries, while resulting in improved performance on different metrics, primarily in contexts where aggregation is present and the variability allows for some model-internal wisdom of the crowd. This mixed picture suggests that when it comes to drawing on ensembles of LLMs, the cost of running multiple iterations at higher temperature may be outweighed by the partial improvements in performance, as is shown in our context of personality item correlation prediction.

Second, while SurveyBot3000 (Hommel & Arslan 2024) has shown strong performance on all our metrics, the fact that it was trained on what is our test set (Condon et al. 2017) makes any conclusion as to its actual performance based on our data not possible. However, the data presented in Hommel & Arslan (2024) show the model's ability to predict the correlations between items, though direct intercomparisons are less straightforward.

Supplementary Figure Captions

Supplementary Figure 1

Mean Prediction Error by Condition

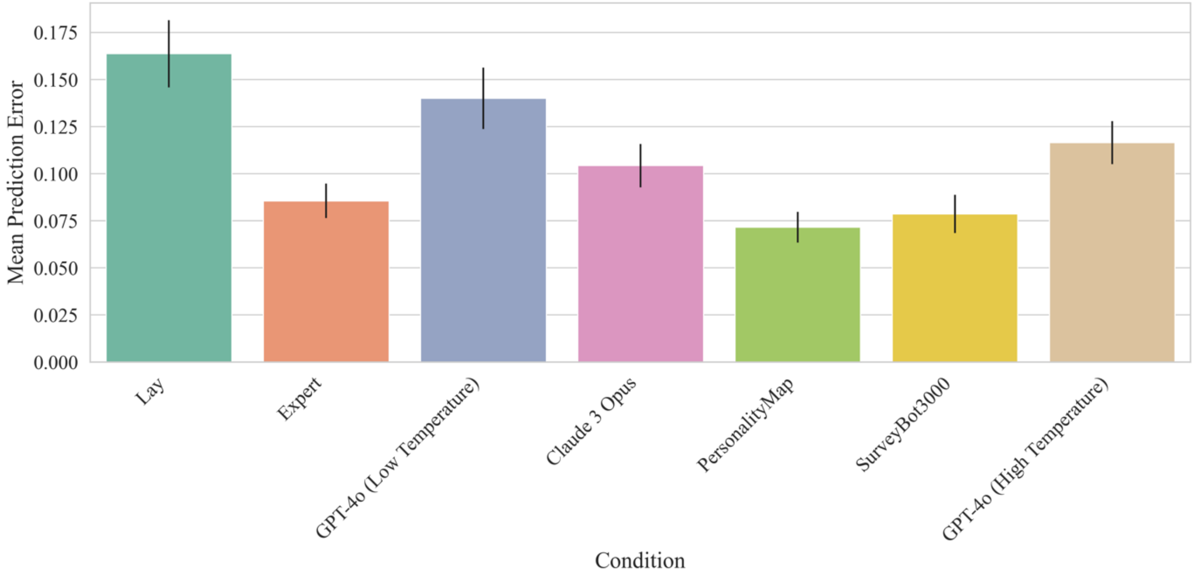

Note. Bar chart with 95% confidence intervals, showing that PersonalityMap, SurveyBot3000, and experts outperform lay people and GPT-4o. Lower bars indicate superior performance.

Supplementary References

1. Magnolia-psychometrics. (n.d.). Synthetic correlations [Web application]. Hugging Face. https://huggingface.co/magnolia-psychometrics/synthetic-correlations
