# Supplementary material for: AI can outperform humans in predicting correlations between personality items
Source: Commun Psychol. 2025 Feb 12;3:23. doi: 10.1038/s44271-025-00205-w (PMC11822111; doi:10.1038/s44271-025-00205-w)
Supplement: Supplementary file 3 — Reporting Summary [file 44271_2025_205_MOESM3_ESM.pdf]

nature portfolio

Corresponding author(s): Philipp Schoenegger

Last updated by author(s): Jan 3, 2025

Reporting Summary

Nature Portfolio wishes to improve the reproducibility of the work that we publish. This form provides structure for consistency and transparency in reporting. For further information on Nature Portfolio policies, see our Editorial Policies and the Editorial Policy Checklist.

Statistics

For all statistical analyses, confirm that the following items are present in the figure legend, table legend, main text, or Methods section.

n/a	Confirmed	
☐	☒	The exact sample size (*n*) for each experimental group/condition, given as a discrete number and unit of measurement
☐	☒	A statement on whether measurements were taken from distinct samples or whether the same sample was measured repeatedly
☐	☒	The statistical test(s) used AND whether they are one- or two-sided<br>*Only common tests should be described solely by name; describe more complex techniques in the Methods section.*
☐	☒	A description of all covariates tested
☐	☒	A description of any assumptions or corrections, such as tests of normality and adjustment for multiple comparisons
☐	☒	A full description of the statistical parameters including central tendency (e.g. means) or other basic estimates (e.g. regression coefficient) AND variation (e.g. standard deviation) or associated estimates of uncertainty (e.g. confidence intervals)
☐	☒	For null hypothesis testing, the test statistic (e.g. *F*, *t*, *r*) with confidence intervals, effect sizes, degrees of freedom and *P* value noted<br>*Give P values as exact values whenever suitable.*
☒	☐	For Bayesian analysis, information on the choice of priors and Markov chain Monte Carlo settings
☒	☐	For hierarchical and complex designs, identification of the appropriate level for tests and full reporting of outcomes
☐	☒	Estimates of effect sizes (e.g. Cohen's *d*, Pearson's *r*), indicating how they were calculated

Our web collection on statistics for biologists contains articles on many of the points above.

Software and code

Policy information about availability of computer code

Data collection	Custom Python code (version 3.10.5) was used with the OpenAI and Anthropic APIs for collecting AI model responses. GuidedTrack and Positly were used for human participant data collection. Data processing utilized pandas (version 1.5.3).
Data analysis	Custom Python code (version 3.10.5) was used for data analysis, utilizing pandas (1.5.3), scipy (1.12.0), numpy (1.26.4), statsmodels (0.14.0), pingouin (0.5.4), matplotlib (3.7.2), and seaborn (0.11.0).

For manuscripts utilizing custom algorithms or software that are central to the research but not yet described in published literature, software must be made available to editors and reviewers. We strongly encourage code deposition in a community repository (e.g. GitHub). See the Nature Portfolio guidelines for submitting code & software for further information.

Data

Policy information about availability of data

All manuscripts must include a data availability statement. This statement should provide the following information, where applicable:

- Accession codes, unique identifiers, or web links for publicly available datasets
- A description of any restrictions on data availability
- For clinical datasets or third party data, please ensure that the statement adheres to our policy

The data and code generated for this study are available in the Open Science Framework (OSF) repository at https://osf.io/kzgy7/files/osfstorage.

Human research participants

Policy information about studies involving human research participants and Sex and Gender in Research.

Reporting on sex and gender	The study reports demographic information for both lay participants and academic experts. For lay participants, 55.91% of participants were men and 44.09% were women. For academic experts, 52.21% were men and 46.32% were women. The study collected this as demographic information from participants' self reports.
Population characteristics	For lay participants: Mean age was 46.35 years (SD=11.83).<br>For academic experts: Mean age was 33.86 years (SD=8.12). 36% were professors, 20% had completed a PhD without being a professor, and 44% were graduate students.
Recruitment	Lay participants were recruited via the Positly.com platform, an online research subject aggregation platform. They were paid $1.80 each for participation (averaging to approximately $8.40 per hour).<br>Academic experts were recruited from academic email lists like SJDM as well as social media such as LinkedIn and X/Twitter. They received a $5 gift card with the option to donate the amount to charity instead.
Ethics oversight	We complied with all ethical regulations for human subjects research. Research protocols were approved by the Institutional Review Board at New York University. Participants gave informed consent to participate in all experiments. We did not use deception.

Note that full information on the approval of the study protocol must also be provided in the manuscript.

Field-specific reporting

Please select the one below that is the best fit for your research. If you are not sure, read the appropriate sections before making your selection.

☐ Life sciences ☒ Behavioural & social sciences ☐ Ecological, evolutionary & environmental sciences

For a reference copy of the document with all sections, see nature.com/documents/nr-reporting-summary-flat.pdf

Behavioural & social sciences study design

All studies must disclose on these points even when the disclosure is negative.

Study description	This is a quantitative comparative study. It compares the performance of AI models (large language models and a specialized AI system) against lay people and academic experts in predicting correlations between personality trait items.
Research sample	The study includes multiple samples:<br><br>254 lay participants recruited via Positly.com platform (mean age 46.35 years, SD=11.83, 56% male)<br>272 academic experts (graduate students, postdocs, or professors in psychology/behavioral science; mean age 33.86 years, SD=8.12, 52% male)<br>AI models: GPT-4o, Claude 3 Opus, and PersonalityMap
Sampling strategy	For human participants, convenience sampling was used. The sample size was determined through a power analysis, aiming for 245 question pairs to detect a small-to-medium effect size (f=0.225) with 80% power at α=0.05 for a one-way ANOVA. To account for partial completions, they aimed to recruit 250 lay participants and 200 experts.<br>For AI models, three runs were collected for each LLM (GPT-4o and Claude 3 Opus) and one run for PersonalityMap due to its deterministic nature.<br>The final dataset included 249 personality item pairs, exceeding the minimum required for the planned analyses.
Data collection	Data was collected through online platforms:<br><br>Lay participants: Used GuidedTrack.com platform to complete the correlation estimation task<br>Experts: Completed the task via an online survey<br>AI models: Queried through respective APIs (GPT-4o and Claude 3 Opus) or proprietary system (PersonalityMap)<br><br>Participants estimated correlations using a slider ranging from -1 to +1 in increments of 0.02. AI models provided numerical predictions.<br>The researchers were not present during data collection and were blind to individual responses during the collection phase.
Timing	The lay and expert samples were collected in March 2024.
Data exclusions	No exclusions.
Non-participation	No participants dropped out.

Randomization	Participants were not allocated into experimental groups in the traditional sense. However, lay participants and experts were randomly assigned 30 item pairs from the total set of 249 pairs to evaluate. For AI models, all 249 item pairs were evaluated, with LLMs providing three estimates per pair and PersonalityMap providing one estimate per pair.

Reporting for specific materials, systems and methods

We require information from authors about some types of materials, experimental systems and methods used in many studies. Here, indicate whether each material, system or method listed is relevant to your study. If you are not sure if a list item applies to your research, read the appropriate section before selecting a response.

Materials & experimental systems

n/a	Involved in the study
☒	☐ Antibodies
☒	☐ Eukaryotic cell lines
☒	☐ Palaeontology and archaeology
☒	☐ Animals and other organisms
☒	☐ Clinical data
☒	☐ Dual use research of concern

Methods

n/a	Involved in the study
☒	☐ ChIP-seq
☒	☐ Flow cytometry
☒	☐ MRI-based neuroimaging
