# Supplementary material for: AI can outperform humans in predicting correlations between personality items
Source: Commun Psychol. 2025 Feb 12;3:23. doi: 10.1038/s44271-025-00205-w (PMC11822111; doi:10.1038/s44271-025-00205-w)
Supplement: Supplementary file 1 — Transparent Peer Review file [file 44271_2025_205_MOESM1_ESM.pdf]

nature portfolio

Peer Review File

AI can outperform humans in predicting correlations between personality items

Corresponding Author: Dr Phillip Schoenegger

This file contains all editorial decision letters in order by version, followed by all author rebuttals in order by version.

Version 0:

Decision Letter:

** Please ensure you delete the link to your author homepage in this e-mail if you wish to forward it to your coauthors **

Dear Dr Schoenegger,

Thank you for your patience during the peer-review process. Your manuscript titled "Can AI Understand Human Personality? - Comparing Human Experts and AI Systems at Predicting Personality Correlations" has now been seen by 3 reviewers, whose comments are appended below. You will see that they find your work of some potential interest. However, they have raised quite substantial concerns that must be addressed. In light of these comments, we cannot accept the manuscript for publication, but would be interested in considering a revised version that fully addresses these serious concerns.

We hope you will find the Reviewers' comments useful as you decide how to proceed. Should additional work allow you to address these criticisms, we would be happy to look at a substantially revised manuscript. If you choose to take up this option, please highlight all changes in the manuscript text file, and provide a detailed point-by-point reply to the reviewers.

Please bear in mind that we will be reluctant to approach the reviewers again in the absence of substantial revisions.

Editorially, we consider the manuscript to have significant potential but also note several critical areas that must be addressed in revision. First, the exploration of variability in AI outputs needs to be deepened, particularly by examining different model parameters and comparing the performance of PersonalityMap with other specialized models, such as SurveyBot3000.

Second, the manuscript lacks sufficient integration with existing literature on AI's role in personality assessment, which is essential for situating the study within the broader scientific context. While the pre-registration of hypotheses is commendable, it is insufficiently clear what gave rise to these hypotheses. Please provide a more robust rationale for your predictions and consider expanding your analyses to ensure the findings are generalizable beyond the specific conditions tested. We recommend that additional analyses are likewise preregistered. Please thoroughly review relevant research and clearly articulate how your work confirms, advances or contrasts with prior findings.

The discussion section must be expanded to connect the findings to the broader literature and address limitations comprehensively; you may include some speculation on the practical implications of the research, but this should be kept to a minimum.

I am attaching a checklist that details critical reporting requirements for the revised manuscript. Please attend to each item and ensure your manuscript is fully compliant. We are requesting that your manuscript aligns with these requirements as this facilitates the evaluation of your manuscript, reducing delays in re-review and potential future acceptance. If your revised manuscript is not aligned with these requests on major issues, such as those concerning statistics, it may be returned to you for further revisions without re-review. Additional information can be found in our style and formatting guide Communications Psychology formatting guide.

If the revision process takes significantly longer than five months, we will be happy to reconsider your paper at a later date, provided it still presents a significant contribution to the literature at that stage.

We would appreciate it if you could keep us informed about an estimated timescale for resubmission, to facilitate our planning.

We are committed to providing a fair and constructive peer-review process. Please do not hesitate to contact us if you wish to discuss the revision in more detail.

Please use the following link to submit your
- revised manuscript,
- point-by-point response to the referees' comments,
- cover letter (as a separate document),
- the Editorial Policy Checklist (see below),
- the Reporting Summary (see below), and
- the completed Editorial Request Table (attached):

Link Redacted

** This url links to your confidential home page and associated information about manuscripts you may have submitted or be reviewing for us. If you wish to forward this email to co-authors, please delete the link to your homepage first **

Thank you for the opportunity to review your work.

Best regards,

Anna-Lena Schubert

Anna-Lena Schubert, PhD
Editorial Board Member
Communications Psychology
orcid.org/0000-0001-7248-0662

REVIEWER EXPERTISE:
Reviewer #1 personality psychology / machine learning
Reviewer #2 personality psychology / machine learning
Reviewer #3 personality psychology / machine learning

REVIEWER REPORTS:

Reviewer #1 (Remarks to the Author):

Review of COMMSPSYCHOL-24-0353-T: "Can AI Understand Human Personality? Comparing Human Experts and AI Systems at Predicting Personality Correlations"

Regarding the stated reviewer guidelines, (a) the article presents an original study, (b) the data and analysis are technically sound and appropriate for the research question, (d) the paper provides strong evidence for its conclusions, and (d) the study question is important to scientists in the specific sub-field of psychology.

• What are the major claims of the paper?

The paper compares and contrasts the abilities of LLMs, lay people, and academic experts to estimate the correlations between personality item pairs.

• Will the paper be of interest to others in the field?

Yes, the paper should generate interest both within and beyond psychology.

• Will the paper influence thinking in the field?

Given that AI and LLMs are hot topics in psychology, the present work has the potential to be a touchstone for the field.

• Are the claims convincing? If not, what further evidence is needed?

Yes, the claims are fairly convincing.

• Have they provided sufficient methodological detail that the experiments could be reproduced?

Yes, the authors provide sufficient methodological detail and documentation, which allow the average reader to be able to reproduce the results.

• Is the statistical analysis of the data sound?

Yes.

• Are there any published articles that compromise scientific advance?

I'm assuming that this awkwardly phrased question is asking whether there are recently published papers that make the present one obsolete in some sense, which may be the wrong interpretation. In any case, I'm unaware of any published paper that otherwise compromises the novelty of what the present paper is striving to achieve.

• Are there other experiments that would strengthen the paper further? How much would they improve it, and how difficult are they likely to be?

No.

• Are the claims appropriately discussed in the context of previous literature?

It appears that the psychological literature relevant to this work has been adequately cited and accurately summarized. Nevertheless, readers might benefit if the authors made a clearer effort in discussing and distinguishing between domain-specific versus domain-general intelligence. This distinction may be particularly important because most LLMs were designed with a fairly domain-specific task in mind (i.e., predicting the "best" or "most appropriate" next word or phrase in an English sentence), yet LLMs are now able to accomplish several domain-general tasks that, at least on a surface level, appear to require some amount of general intelligence.

• Are there any special ethical concerns arising from the use of animals or human subjects?

No/none.

• If the manuscript is unacceptable in its present form, does the study seem sufficiently promising that the authors should be encouraged to consider a resubmission in the future?

N/A

• Is the manuscript clearly written? If not, how could it be made more accessible?

The manuscript is clearly written; however, the Discussion section's Limitations subsection could be improved by including a paragraph on constraints on generality (Simons et al., 2017), especially given what appears to be a fairly W.E.I.R.D. (Henrich et al., 2010) sample. In addition, readers would benefit is a brief Conclusions paragraph were added to the end of the Discussion section.

• Could the manuscript be shortened to aid communication of the most important findings?

Although the manuscript could be shortened, I believe doing so might be more harmful than beneficial, given the complexity of some of the methods and analyses.

• Have the authors done themselves justice without overselling their claims?

The authors do not appear to be over-selling or mischaracterizing their research findings.

• Have they been fair in their treatment of previous literature?

The authors have been reasonable and fair in covering the prior relevant literature.

• Should the authors be asked to provide further data or methodological information to help others replicate their work? (Such data might include study materials, detailed protocols or mathematical derivations).

It appears that the author have included the necessary information to allow others to successfully reproduce or critically re-analyze their work.

External References

Henrich, J., Heine, S. J., & Norenzayan, A. (2010). The weirdest people in the world? Behavioral and Brain Sciences, 33, 61–83. https://doi.org/10.1017/S0140525X0999152X

Simons, D. J., Shoda, Y., & Lindsay, D. S. (2017). Constraints on generality (COG): A proposed addition to all empirical papers. Perspectives on Psychological Science, 12(6), 1123–1128. https://doi.org/10.1177/1745691617708630

Reviewer #2 (Remarks to the Author):

I have been asked to review this manuscript. For transparency, please note that I have expertise in psychometrics and personality research. I have no conflicts of interest in reviewing this paper, nor am I an author of any of the papers I referenced in my review.

In the submitted manuscript, the authors report on a study investigating the capabilities of a fine-tuned AI model and general-purpose AI models for predicting the magnitude of statistical associations between 249 pairs of Likert-type-scale personality items from the SAPA Personality Inventory. Using layperson and expert ratings as benchmarks, they show that the performance of the PersonalityMap neural network is similar to the median rating of a layperson or expert sample and superior to general-purpose LLMs. I see the contribution of the study in its clear practical scope, evaluating different methods for deriving (cost-effective) AI-based estimates of population parameters for which researchers would otherwise need to collect test response data from human participants. In contrast, I do not think the manuscript makes a theoretical contribution to personality research. Therefore, I believe that the analyses related to Research Question 2 provide the core contribution of the present manuscript, while the analyses related to Research Question 1 seem, frankly, unnecessary.

While the work presented in the manuscript is timely and likely to be of interest to personality researchers, I suspect that its relevance is limited by the lack of demonstrated generalizability of the findings. In its current form, I think the manuscript falls short of providing convincing evidence on how best to approximate personality item correlations, and so far seems to offer only what could be considered anecdotal evidence, or perhaps proof of concept, that correlations between (self-report) personality items could be reasonably approximated by human raters or AI tools. This makes me question whether the paper in its current form could make a sufficiently meaningful contribution to the literature. Also, based on my reading of the manuscript, the authors are not framing their study as a proof of concept, but are aiming for more general conclusions.

Given that data collection using LLMs and other AI models is both cheap and straightforward, I think it would be reasonable to significantly expand the analyses to foster evidence of generalizability, concrete examples of which I outline below in comments 1-2. Given the practical scope of the work, I also think the potential impact of the paper can be significantly increased by extending the analysis to include SurveyBot3000, which is another fine-tuned model for predicting personality item correlations, as I point out in comment 3. In addition, I make several suggestions for additional improvements to the manuscript in my comments 4-7.

Major points:

(1) General barriers to generalizability: The study is limited to a single instrument (i.e., the SAPA Inventory) and thus to a single item type (i.e., self-descriptive statements) and response scale (i.e., 6-point scale). It is also limited to a single domain of interindividual differences (i.e., broad personality traits). It is limited to correlations based on self-report measures. It is limited to concurrent correlations from a cross-sectional assessment. It uses a limited sample size of 249 correlation coefficients from a single population.

(2) Specific barriers to the generalizability of the conclusion that PersonalityMap outperforms general-purpose LLMs: The LLMs were limited to using a specific approach (e.g., setting the temperature to 0 and using a strategy of extensive prompt augmentation as described in Appendix B). What if the LLMs were given the same instructions as the laypersons without extensive prompting? What if LLMs were used with higher temperature parameters and then aggregated over more runs? What if LLMs were asked to evaluate multiple pairs of items at once instead of one at a time? These examples show that there are many different approaches that could be used alternatively, some of which may or may not perform better or worse. So how certain can we really be that personalityMap is generally better than LLMs at predicting correlations between personality items?

(3) In their preprint, Hommel & Arslan (2024) have recently introduced a specialized AI model (SurveyBot3000) trained to predict correlations between personality items, and provide an openly accessible app here that the authors are certainly familiar with https://huggingface.co/spaces/magnolia-psychometrics/synthetic-correlations. I think an important insight for personality researchers would be whether personalityMap or SurveyBot3000 (or any other AI model) is better at predicting correlations in your study. Needless to say, I would also give this recommendation to Hommel & Arslan (2024) and suggest that they include PersonalityMap in a revision of their preprint.

Additional points:

(4) The methodology section is missing some important information. What sample of participants were the correlation coefficients derived from? What was the population, what was the sample size? This would provide important context for the results, since imprecision or bias in estimating the true correlations places an upper bound on the performance that can be achieved.

(5) Id suggest calculating the ICC(2,k) https://doi.org/10.1037/0033-2909.86.2.420 as an estimate of the interrater reliability of

the mean rating for a sample of k judges, which would give an indication of whether adding additional judges would increase reliability. For example, this statistic is implemented in R packages such as psych by William Revelle. Since this is not currently reported in the manuscript, I have no way to be sure that 3 runs are sufficient to achieve a sufficient reliability of, for example, ICC(2,k) > .95. If the ICC(2,k) turns out to be below that for GPT-4o or Claude 3, then more runs should be added to these methods to potentially improve their performance.

(6) I think that predicting the (relative) pattern of correlation coefficients (as done in H2b) is much more informative about true performance than predicting the absolute correlation coefficients (as done in H2a), especially given that the correlations were derived from self-report data and are unlikely to represent "true" correlations between different experiential or behavioral patterns. Self-reports are likely to be inflated by various sources of systematic measurement error that affect the general assessment of correlation estimates, such as biases related to global self-report (e.g., known as common method biases; Podsakoff et al., 2024 https://doi.org/10.1146/annurev-orgpsych-110721-040030). Note that correlations derived from multimethod data tend to be much lower. Thus, could the better performance in predicting absolute correlation coefficients as found in self-reports also be explained by the reproduction of design-specific biases associated with the use of self-reports (rather than true signal)? I suggest that this aspect be discussed more critically and that more weight be given to the results for the relative pattern of correlation coefficients in the conclusions, which suggest that the median ratings of laypersons and experts and the ratings by PersonalityMap seem to be on a par in terms of predictive performance.

(7) In a similar vein, from a descriptive perspective, it would be useful to briefly describe the distribution of scores for the different methods and compare them to the distribution of correlation coefficients. Also, Figure 2 only shows the absolute prediction errors, not whether the methods tend to over- or underestimate the correlations.

Reviewer #3 (Remarks to the Author):

Can AI Understand Human Personality? - Comparing Human Experts and AI Systems at Predicting Personality Correlations

The paper explores the ability of LLMs (GPT-4o and Claude 3 Opus) and a specialized model called PersonalityMap, to predict correlations between personality questionnaire items. The study compares these model-based predictions with those made by laypeople and academic experts. The findings reveal that while ML models, especially PersonalityMap, outperform most individual humans, aggregate expert predictions still match or surpass the performance of LLMs. While the topic is of scientific interest and the manuscript has the potential to make a novel contribution, there are several major issues that should be addressed prior to publication.

Major Concerns

1) Motivation and Integration with Prior Work

While the overall question about ML models' "understanding" of human personality is important, the authors boil this problem down to a much more narrow question about the prediction of correlations between questionnaire items. In doing so, the authors miss the opportunity to (1) sufficiently motivate their research and (2) tie their work in with prior research on the topic. Specifically, there is a whole stream of research investigating different aspects of LLM's understanding of personality, including (1) their ability to infer personality from text and conversational interactions, (2) simulate personality-congruent responses, and 3) utilize personality to adaptively interact with humans.

Relevant sources:
Peters, H., & Matz, S. (2024). Large language models can infer psychological dispositions of social media users. PNAS Nexus, pgae231. https://doi.org/10.1093/pnasnexus/pgae231

Zhang, Tianyi, Antonis Koutsoumpis, Janneke K. Oostrom, Djurre Holtrop, Sina Ghassemi, and Reinout E. de Vries. "Can Large Language Models Assess Personality from Asynchronous Video Interviews? A Comprehensive Evaluation of Validity, Reliability, Fairness, and Rating Patterns." IEEE Transactions on Affective Computing, 2024, 1–16. https://doi.org/10.1109/TAFFC.2024.3374875.

Peters, H., Cerf, M., & Matz, S. C. (2024). Large Language Models Can Infer Personality from Free-Form User Interactions (arXiv:2405.13052). arXiv. https://doi.org/10.48550/arXiv.2405.13052

Pellert, Max, Clemens M. Lechner, Claudia Wagner, Beatrice Rammstedt, and Markus Strohmaier. "AI Psychometrics: Assessing the Psychological Profiles of Large Language Models Through Psychometric Inventories." Perspectives on Psychological Science, January 2, 2024, 17456916231214460. https://doi.org/10.1177/17456916231214460.

Jiang, Guangyuan, Manjie Xu, Song-Chun Zhu, Wenjuan Han, Chi Zhang, and Yixin Zhu. "Evaluating and Inducing Personality in Pre-Trained Language Models." Advances in Neural Information Processing Systems 36 (December 15, 2023): 10622–43.

Dorner, Florian E., Tom Sühr, Samira Samadi, and Augustin Kelava. "Do Personality Tests Generalize to Large Language Models?" arXiv:2311.05297 [Cs], November 9, 2023. https://doi.org/10.48550/arXiv.2311.05297.

Matz, S. C., Teeny, J. D., Vaid, S. S., Peters, H., Harari, G. M., & Cerf, M. (2024). The potential of generative AI for

personalized persuasion at scale. Scientific Reports, 14(1), 4692. https://doi.org/10.1038/s41598-024-53755-0

I suggest the authors thoroughly review the previous literature and then motivate their own work by explaining how it adds to prior research in this field. Additionally, the authors should carefully justify why it is important to understand the relationships between personality items, what their research would reveal about the abilities of AI models, and what theoretical or practical implications their results could have.

2) Justification of Hypotheses

The fact that the authors pre-registered their hypotheses is applaudable. However, since there is no theoretical grounding or rationale given for the hypotheses the research still strikes me as, overall, exploratory in nature. The authors should attempt to justify their hypotheses more thoroughly.

3) Variability in Model Outputs and Wisdom of Crowds Effects

In the current setup, summary statistics are not very meaningful for ML models, given the low number of LLM runs and the decision to use a temperature of T=0, which reduces the variability in outputs. Therefore, Table 1 does not seem to add much to the overall story of the manuscript. More importantly, the findings regarding the relative accuracy of median human estimates beg the question as to whether a similar effect would be observed for LLM-inferred scores. I suggest the authors run an additional experiment where LLMs repeatedly generate scores over a larger number of iterations with a higher temperature value (e.g., the default of T=1), and analyze how aggregate scores compare to human aggregates and Personal ityMap.

4) Discussion Section

The discussion session is lacking in several ways. First, the discussion mostly restates the results but does not sufficiently tie the current research in with previous work and the broader literature. Second, the manuscript ends very abruptly with a very short Limitations section. I recommend expanding the Limitations section with material currently placed in footnotes, as well as concerns raised by reviewers. Finally, a separate section on practical implications and a short conclusion section would round out the paper.

Minor Concerns

5) "Our samples were willing to answer more questions than anticipated" (p. 9) - I assume you meant to say "participants".

6) Throughout the manuscript the authors frequently refer to ML modes as "machines" in contrast to "humans". This dichotomy has a pop-cultural connotation that the authors might want to avoid. Why not call them "ML models"?

7) The footnote on page 14 is rather long and a bit confusing. Maybe it would make sense to add some context and discuss these points in the Limitations section?

8) "However, it is also possible that, by a fluke of what items the humans were randomly assigned to predict, some item correlations might arise more in the human sample than in the machine sample" (p.14) - I assume you could verify empirically whether this is indeed the case.

9) Tables should be understandable in isolation. Please make sure to include sufficient information in the notes. For example, it was not clear in Table 5 what the correlations were referring to.

10) Why were CIs computed on z scores and not on the correlation coefficients themselves in Table 5? The latter seems more common and more intuitive. Relatedly, Figure 5 would be much more intuitive if it showed correlation coefficients instead of z scores.

11) The analysis on bucketed prediction errors (Hypothesis 2c) does not seem to add much above and beyond the previous analyses. The authors might want to consider moving it to the SI.

12) "Our results suggest that current AI models are roughly as good as, if not better, than human experts in predicting correlations amongst human personality traits." (p. 25) - The wording is imprecise. The predictions are not about relationships between traits but between questionnaire items. A similar concern applies to the same statement on page 3.

13) "To make an analogy, imagine if biologists could only ever conduct research in human bodies (in vivo) without the ability to do experiments in vitro." (p. 28) - This analogy confuses more than it clarifies.

Conclusion

While the manuscript reveals novel insights on the ability of ML models to represent personality constructs, there are several major issues that need to be addressed. Most importantly, the authors need to better motivate their research question and integrate their work with the existing literature. The authors should also provide justifications for their hypotheses and conduct additional experiments to examine the quality of aggregate model-based predictions. Finally, the discussion section should be expanded to tie the findings back to the prior literature, address limitations more comprehensively, and outline the practical implications of the study. Additional revisions to the manuscript's language, clarity, and table presentation would be helpful as well.

Given these substantial concerns, I recommend a major revision of the manuscript.

EDITORIAL POLICIES

We ask that you ensure your manuscript complies with our editorial policies and reporting requirements.

To that end, we require revised manuscripts to be accompanied by two completed items: a reporting summary that collects information on study design and procedure, and an editorial policy checklist that verifies compliance with all required editorial policies

- Nature Research Reporting Summary
- Editorial Policy Checkl ist

All points on the policy checklist must be addressed. Your revised manuscript can only be sent back to the referees if these checklists are completed and uploaded with the revision.

Notes: If you have submitted a Stage 1 Registered Report, Review, Primer, Comment, or Perspective you do not need to submit these forms. If you have already submitted these forms, you may disregard this request.

* TRANSPARENT PEER REVIEW: Communications Psychology uses a transparent peer review system. This means that we publish the editorial decision letters including Reviewers' comments to the authors and the author rebuttal letters online as a supplementary peer review file. However, on author request, confidential information and data can be removed from the published reviewer reports and rebuttal letters prior to publication. If your manuscript has been previously reviewed at another journal, those Reviewers' comments would not form part of the published peer review file.

** Visit Nature Research's author and referees' website at www.nature.com/authors for information about policies, services and author benefits**

Communications Psychology is committed to improving transparency in authorship. As part of our efforts in this direction, we are now requesting that all authors identified as 'corresponding author' create and link their Open Researcher and Contributor Identifier (ORCID) with their account on the Manuscript Tracking System prior to acceptance. ORCID helps the scientific community achieve unambiguous attribution of all scholarly contributions. You can create and link your ORCID from the home page of the Manuscript Tracking System by clicking on 'Modify my Springer Nature account' and following the instructions in the link below. Please also inform all co-authors that they can add their ORCIDs to their accounts and that they must do so prior to acceptance.
https://www.springernature.com/gp/researchers/orcid/orcid-for-nature-research

For more information please visit http://www.springernature.com/orcid

If you experience problems in linking your ORCID, please contact the Platform Support Helpdesk.

Version 1:

Decision Letter:

** Please ensure you delete the link to your author homepage in this e-mail if you wish to forward it to your coauthors **

Dear Dr Schoenegger,

Your manuscript titled "Can AI Understand Human Personality? - Comparing Human Experts and AI Systems at Predicting

Personality Correlations" has now been seen by our reviewers, whose comments appear below. In light of their advice I am delighted to say that we are happy, in principle, to publish a suitably revised version in Communications Psychology.

We therefore invite you to revise your paper one last time to address the remaining concerns of our reviewers and a list of editorial requests. At the same time we ask that you edit your manuscript to comply with our format requirements and to maximise the accessibility and therefore the impact of your work. Please note that we will not be able to proceed towards publication unless all editorial requests, including the prioritized list of reviewer concerns below, are suitably addressed.

EDITORIAL REQUESTS:

Editorially, we ask that you limit speculation regarding the potential use of the models in comparison to more established alternatives. In addition, we ask that you improve the justification for the precise choices of analysis that you implemented to test the wider hypotheses. Additional justifications for the hypothesis or study themselves are not necessary. We do see the value in basing inferences regarding the comparison of correlations based on Z-values instead of raw correlation coefficients; however, adding 95% CIs around the raw correlation coefficients will still provide useful information for many readers.

Please review our specific editorial comments and requests regarding your manuscript in the attached "Editorial Requests Table". Please outline your response to each request in the right hand column. Please upload the completed table with your manuscript files as a Related Manuscript file.

If you have any questions or concerns about any of our requests, please do not hesitate to contact me.

SUBMISSION INFORMATION:

In order to accept your paper, we require the files listed here https://www.nature.com/documents/commsj-file-checklist.pdf .

OPEN ACCESS:

Communications Psychology is a fully open access journal. Articles are made freely accessible on publication. For further information about article processing charges, open access funding, and advice and support from Nature Research, please visit https://www.nature.com/commspsychol/open-access

At acceptance, you will be provided with instructions for completing the open access licence agreement on behalf of all authors. This grants us the necessary permissions to publish your paper. Additionally, you will be asked to declare that all required third party permissions have been obtained, and to provide billing information in order to pay the article-processing charge (APC).

* TRANSPARENT PEER REVIEW: Communications Psychology uses a transparent peer review system. On author request, confidential information and data can be removed from the published reviewer reports and rebuttal letters prior to publication. If you are concerned about the release of confidential data, please let us know specifically what information you would like to have removed. Please note that we cannot incorporate redactions for any other reasons.

* CODE AVAILABILITY: All Communications Psychology manuscripts must include a section titled "Code Availability" at the end of the methods section. We require that the custom analysis code supporting your conclusions is made available in a publicly accessible repository at this stage; please choose a repository that generates a digital object identifier (DOI) for the code; the link to the repository and the DOI must be included in the Code Availability statement. Publication as Supplementary Information will not suffice.

* DATA AVAILABILITY:
All Communications Psychology manuscripts must include a section titled "Data Availability" at the end of the Methods section. More information on this policy, is available in the Editorial Requests Table and at http://www.nature.com/authors/policies/data/data-avai labil ity-statements-data-citations.pdf.

Please use the following link to submit the above items:
Link Redacted
** This url links to your confidential home page and associated information about manuscripts you may have submitted or be reviewing for us. If you wish to forward this email to co-authors, please delete the link to your homepage first **

We hope to hear from you within four weeks; please let us know if you need more time.

Best regards,

Marike Schiffer

on behalf of Anna-Lena Schubert

Marike Schiffer, PhD
Chief Editor
Communications Psychology

Anna-Lena Schubert, PhD
Editorial Board Member
Communications Psychology
orcid.org/0000-0001-7248-0662

REVIEWERS' COMMENTS:

Reviewer #2 (Remarks to the Author):

In my previous review, I noted several concerns with the framing of the manuscript and suggested potential ways to refocus it on the practical implications of collecting synthetic estimates of correlations between personality items. I also commented on strategies to strengthen the generalizability of the findings or to clarify related limitations. I am pleased to note that the authors have been exceptionally responsive, carefully considering all of this feedback and effectively addressing my concerns. I have no further comments at this time.

Reviewer #3 (Remarks to the Author):

The authors have made veritable attempts to address the suggestions from the previous round of reviews. However, several problems remain that need to be solved before I can recommend publication.

Re Comment 1. The authors have integrated the previous literature and point out how their work adds to it. However, many of the newly introduced arguments strike me as half-baked. For example, the authors claim that "There are many reasons that it can be useful to understand the relationships between self-reported personality items, including to test hypotheses (e.g., that people with anxiety also often have depression), to develop scales (e.g., to identify items that could help measure a trait such as narcissism), and to generate new hypotheses (e.g., by exploring what items are predictive reporting being unhappy with their relationships).", but the examples do not relate well to personality psychology. Especially the example about anxiety and depression seems out of scope given its association with clinical psychology. Many of the other points are not very convincing either, as empirical item correlations are easily obtainable in practice, which considerably limits the utility of model-based predictions. I understand that predictions of item correlations can be helpful in test development, but it is unclear to me how this would translate into the much broader claims made by the authors on page 7 (e.g., "If general models like LLMs could achieve high accuracy, they might enable applications in hiring, healthcare, and personalized marketing, reducing reliance on extensive questionnaires or expert input while adapting quickly to new contexts"). The authors should either scale back their claims or explain exactly how predicted item correlations would be used in practice.

Re Comment 2.2. The authors have added additional explanations but still do not include a stringent justification of the hypotheses. I would like to see improvements made to this section.

Re Comment 10: The authors responded to my suggestion to show correlation coefficients instead of z-scores: "We preregistered our analysis using Fisher's z-scores to account for what we anticipated would be many conditions with similarly high correlations (e.g., 0.9 and above). In cases like these, z-scores offer a key advantage because the z-transformation stabilises the variance of correlation coefficients, particularly in the upper and lower extremes where correlations approach 1 or -1. This stabilisation enables us to compute confidence intervals (CIs) more accurately and ensures that the CIs remain symmetric, which is harder to achieve when working directly with raw correlations due to their potential skewness. While we understand that this may be less intuitive, we also report the full correlation values in Table 5." I respectfully disagree with this reasoning. The authors could easily report correlation coefficients and test for differences using generally accepted methods such as the Fishers-Z test. If there are distribution-related arguments against this approach, this should be explicitly discussed in the manuscript or SI. The current presentation confuses more than it explains.
New comment: There is a mismatch between the title and the actual question answered in the paper. The title uses the term "personality correlations", which can easily be interpreted as correlations with other constructs and criteria. Given that the paper is concerned with item correlations, a much more narrow question, I would suggest adjusting the title.

Conclusion
Overall, I appreciate the authors' efforts in addressing my previous suggestions. However, as stated above, there is further room for improvement. I would, therefore, recommend another round of revisions.

** Visit Nature Research's author and referees' website at www.nature.com/authors for information about policies, services and author benefits**

Open Access This Peer Review File is licensed under a Creative Commons Attribution 4.0 International License, which permits use, sharing, adaptation, distribution and reproduction in any medium or format, as long as you give appropriate credit to the original author(s) and the source, provide a link to the Creative Commons license, and indicate if changes were made.
In cases where reviewers are anonymous, credit should be given to 'Anonymous Referee' and the source.
The images or other third party material in this Peer Review File are included in the article's Creative Commons license, unless indicated otherwise in a credit line to the material. If material is not included in the article's Creative Commons license and your intended use is not permitted by statutory regulation or exceeds the permitted use, you will need to obtain permission directly from the copyright holder.
To view a copy of this license, visit https://creativecommons.org/licenses/by/4.0/

Reviewer #1

Comment 1: Review of COMMSPSYCHOL-24-0353-T: “Can AI Understand Human Personality? Comparing Human Experts and AI Systems at Predicting Personality Correlations”

Regarding the stated reviewer guidelines, (a) the article presents an original study, (b) the data and analysis are technically sound and appropriate for the research question, (d) the paper provides strong evidence for its conclusions, and (d) the study question is important to scientists in the specific sub-field of psychology.

Comment 2: • What are the major claims of the paper?

The paper compares and contrasts the abilities of LLMs, lay people, and academic experts to estimate the correlations between personality item pairs.

Comment 3: • Will the paper be of interest to others in the field?

Yes, the paper should generate interest both within and beyond psychology.

Comment 4:• Will the paper influence thinking in the field?

Given that AI and LLMs are hot topics in psychology, the present work has the potential to be a touchstone for the field.

Comment 5: • Are the claims convincing? If not, what further evidence is needed?

Yes, the claims are fairly convincing.

Response: Thank you for your assessment! We have also added additional analyses based on other reviewer requests, which should make our conclusions even more convincing!

Comment 6: • Have they provided sufficient methodological detail that the experiments could be reproduced?

Yes, the authors provide sufficient methodological detail and documentation, which allow the average reader to be able to reproduce the results.

Comment 7: • Is the statistical analysis of the data sound?

Yes.

Comment 8: • Are there any published articles that compromise scientific advance?

I'm assuming that this awkwardly phrased question is asking whether there are recently published papers that make the present one obsolete in some sense, which may be the wrong interpretation. In any case, I'm unaware of any published paper that otherwise compromises the novelty of what the present paper is striving to achieve.

Response: We have also expanded our treatment of the literature (including some papers that came out after we submitted this manuscript) to ensure all current literature is properly portrayed with respect to our contribution.

Comment 9: • Are there other experiments that would strengthen the paper further? How much would they improve it, and how difficult are they likely to be?

No.

Response: We would like to point out that in response to other reviewers, we have added two additional AI conditions (a high-temperature version of GPT-4o as well as SurveyBot3000, a model developed for a different paper on a similar topic). Throughout the paper, we have highlighted in yellow the passages that we have revised the most or added throughout the revision.

Comment 10: • Are the claims appropriately discussed in the context of previous literature?

It appears that the psychological literature relevant to this work has been adequately cited and accurately summarized. Nevertheless, readers might benefit if the authors made a clearer effort in discussing and distinguishing between domain-specific versus domain-general intelligence. This distinction may be particularly important because most LLMs were designed with a fairly domain-specific task in mind (i.e., predicting the "best" or "most appropriate" next word or phrase in an English sentence), yet LLMs are now able to accomplish several domain-general tasks that, at least on a surface level, appear to require some amount of general intelligence.

Response: Thank you very much for pressing us on adding this! We have now added a full paragraph early on that sets up this distinction in more detail.

Example: For the purposes of this paper, we distinguish domain-specific (specialised) and domain-general (generalised) models. Both types of machine learning models share the feature

that they are trained with a quite narrow objective such as predicting the next token. However, specialised models, as they are deployed, only take a very specific type of input (such as tabular data, genetic profiles, or a picture) and provide a specific output (such as a statistical value, a health risk profile, or a classification). Contrast this with generalised models like LLMs that, due to their architecture, can operate with a large number of inputs as well as outputs ranging from words to numbers and even images. Not only do LLMs enable a variety of modalities, they are also domain-general in that one can ask them about a puppy's health condition, interest rate projections for Peru, or an interpretation of a regression output table, primarily because of their vast training data. This makes these models fundamentally different from previous artificial intelligence models, raising the question of how well generalised models (LLMs) perform compared to specialised models in a number of specific applications.

Comment 11: • Are there any special ethical concerns arising from the use of animals or human subjects?

No/none.

Comment 12: • If the manuscript is unacceptable in its present form, does the study seem sufficiently promising that the authors should be encouraged to consider a resubmission in the future?

N/A

Comment 13: • Is the manuscript clearly written? If not, how could it be made more accessible?

The manuscript is clearly written; however, the Discussion section's Limitations subsection could be improved by including a paragraph on constraints on generality (Simons et al., 2017), especially given what appears to be a fairly W.E.I.R.D. (Henrich et al., 2010) sample. In addition, readers would benefit is a brief Conclusions paragraph were added to the end of the Discussion section.

Henrich, J., Heine, S. J., & Norenzayan, A. (2010). The weirdest people in the world? Behavioral and Brain Sciences, 33, 61–83. https://doi.org/10.1017/S0140525X0999152X

Simons, D. J., Shoda, Y., & Lindsay, D. S. (2017). Constraints on generality (COG): A proposed addition to all empirical papers. Perspectives on Psychological Science, 12(6), 1123–1128. https://doi.org/10.1177/1745691617708630

Response: Thank you for the idea of adding this - we have now added a more exhaustive paragraph to the limitations section, going over a variety of issues to generalisation like the one

you outlined as well as others.

Example: Another limitation is that our results are also limited by the choice of data we use to test the models and humans on, reducing generalisability. Specifically, we only use a single source to draw our item pairs from (the SAPA Inventory), resulting in a single item type of self-descriptive statements based on self-report in only one mode of assessment (cross-sectional). A further constraint on the generalisability of our findings is that we drew on a W.E.I.R.D. sample (Henrich et al. 2010, Simons et al. 2017). These concerns limit how much we can generalise from our results. It is plausible that different items might lead to different results that may not replicate in different samples. However, as self-report items remain the backbone of contemporary personality psychology, we believe that, at least with respect to the current paradigm, our results should hold up.

Comment 14: • Could the manuscript be shortened to aid communication of the most important findings?

Although the manuscript could be shortened, I believe doing so might be more harmful than beneficial, given the complexity of some of the methods and analyses.

Comment 15: • Have the authors done themselves justice without overselling their claims?

The authors do not appear to be over-selling or mischaracterizing their research findings.

Comment 16: • Have they been fair in their treatment of previous literature?

The authors have been reasonable and fair in covering the prior relevant literature.

Comment 17: • Should the authors be asked to provide further data or methodological information to help others replicate their work? (Such data might include study materials, detailed protocols or mathematical derivations).

It appears that the author have included the necessary information to allow others to successfully reproduce or critically re-analyze their work.

Response: We just wanted to point out that full code and data are available for anyone to download: https://osf.io/kzgy7/files/osfstorage

Reviewer #2

General Comment: I have been asked to review this manuscript. For transparency, please note that I have expertise in psychometrics and personality research. I have no conflicts of interest in reviewing this paper, nor am I an author of any of the papers I referenced in my review.

In the submitted manuscript, the authors report on a study investigating the capabilities of a fine-tuned AI model and general-purpose AI models for predicting the magnitude of statistical associations between 249 pairs of Likert-type-scale personality items from the SAPA Personality Inventory. Using layperson and expert ratings as benchmarks, they show that the performance of the PersonalityMap neural network is similar to the median rating of a layperson or expert sample and superior to general-purpose LLMs. I see the contribution of the study in its clear practical scope, evaluating different methods for deriving (cost-effective) AI-based estimates of population parameters for which researchers would otherwise need to collect test response data from human participants. In contrast, I do not think the manuscript makes a theoretical contribution to personality research. Therefore, I believe that the analyses related to Research Question 2 provide the core contribution of the present manuscript, while the analyses related to Research Question 1 seem, frankly, unnecessary.

While the work presented in the manuscript is timely and likely to be of interest to personality researchers, I suspect that its relevance is limited by the lack of demonstrated generalizability of the findings. In its current form, I think the manuscript falls short of providing convincing evidence on how best to approximate personality item correlations, and so far seems to offer only what could be considered anecdotal evidence, or perhaps proof of concept, that correlations between (self-report) personality items could be reasonably approximated by human raters or AI tools. This makes me question whether the paper in its current form could make a sufficiently meaningful contribution to the literature. Also, based on my reading of the manuscript, the authors are not framing their study as a proof of concept, but are aiming for more general conclusions.

Given that data collection using LLMs and other AI models is both cheap and straightforward, I think it would be reasonable to significantly expand the analyses to foster evidence of generalizability, concrete examples of which I outline below in comments 1-2. Given the practical scope of the work, I also think the potential impact of the paper can be significantly increased by extending the analysis to include SurveyBot3000, which is another fine-tuned model for predicting personality item correlations, as I point out in comment 3. In addition, I make several suggestions for additional improvements to the manuscript in my comments 4-7.

Response: Thank you very much for your clear and actionable comments on our paper and your transparency! On reflection, we agree that our previous manuscript might have overstated the generalisability of our results at times, but hope that the revised manuscript now more accurately reflects the data, in part by not overclaiming the results about personality prediction as a whole based on questionnaire item relationship prediction capabilities. We have also

included SurveyBot3000 into our paper as requested (more on this below at your specific comment). In our revised paper, we have also highlighted in yellow the passages that we reworked the most or that we added during the revisions.

Comment 1: Major points: (1) General barriers to generalizability: The study is limited to a single instrument (i.e., the SAPA Inventory) and thus to a single item type (i.e., self-descriptive statements) and response scale (i.e., 6-point scale). It is also limited to a single domain of interindividual differences (i.e., broad personality traits). It is limited to correlations based on self-report measures. It is limited to concurrent correlations from a cross-sectional assessment. It uses a limited sample size of 249 correlation coefficients from a single population.

Response: We added a paragraph to the limitations section that outlines the concerns raised here (as well as others like the WEIRD sample). Additionally, another reviewer had also raised some generalisability concerns, so we now discuss an even wider set of barriers.

Example: Another limitation is that our results are also limited by the choice of data we use to test the models and humans on, reducing generalisability. Specifically, we only use a single source to draw our item pairs from (the SAPA Inventory), resulting in a single item type of self-descriptive statements based on self-report in only one mode of assessment (cross-sectional). A further constraint on the generalisability of our findings is that we drew on a W.E.I.R.D. sample (Henrich et al. 2010, Simons et al. 2017). These concerns limit how much we can generalise from our results. It is plausible that different items might lead to different results that may not replicate in different samples. However, as self-report items remain the backbone of contemporary personality psychology, we believe that, at least with respect to the current paradigm, our results should hold up.

Comment 2: (2) Specific barriers to the generalizability of the conclusion that PersonalityMap outperforms general-purpose LLMs: The LLMs were limited to using a specific approach (e.g., setting the temperature to 0 and using a strategy of extensive prompt augmentation as described in Appendix B). What if the LLMs were given the same instructions as the laypersons without extensive prompting? What if LLMs were used with higher temperature parameters and then aggregated over more runs? What if LLMs were asked to evaluate multiple pairs of items at once instead of one at a time? These examples show that there are many different approaches that could be used alternatively, some of which may or may not perform better or worse. So how certain can we really be that personalityMap is generally better than LLMs at predicting correlations between personality items?

Response: To address your concern, we have directly followed up on your suggestion, which was also mirrored by Reviewer 3, of adding an LLM condition with high temperature. As budgetary constraints made the addition of Claude 3 Opus at high temperature at much higher cost per token not feasible, we added GPT-4o at T=1 for 30 runs for each item. We find that for most comparisons, the high temperature model performs like the low temperature one, with only

one exception at an aggregate comparison. We outline these results in the ‘Additional Results’ section, as well as in great detail in the Appendix D.

Example: Second, we also ran an additional condition with the same GPT-4o model that we preregistered (gpt-4o-2024-05-13), but at a high temperature (of 1) with 30 runs for each item (which we then take the median of), to further test current frontier model performance at different parameters. Temperature is a hyperparameter for LLMs that controls the randomness of the model's predictions - at higher temperatures models are more "creative", producing more unique and unusual responses. While higher temperatures typically produce worse performance, one strategy to try to improve LLM performance is to generate more responses at higher temperature and then combine them (e.g., through taking the median). Of course, this also comes at greatly increased cost, since the LLM must be run many times. We find that for most of our preregistered comparisons, the high-temperature version of GPT-4o with 30 runs is on par with the low-temperature version at 3 runs. However, we find that in the aggregate analysis, drawing on the more varied predictions from higher temperature leads to improved performance on one of our measures. Specifically, with respect to the correlation between median predicted correlation (across the different runs of the same model) and the empirical correlation, the high-temperature multiple-runs condition of GPT-4o is not statistically different from PersonalityMap, whereas the low-temperature version of GPT-4o was statistically worse. For a full set of results of this model on our preregistered hypotheses, see Appendix D.

Comment 3: (3) In their preprint, Hommel & Arslan (2024) have recently introduced a specialized AI model (SurveyBot3000) trained to predict correlations between personality items, and provide an openly accessible app here that the authors are certainly familiar with https://huggingface.co/spaces/magnolia-psychometrics/synthetic-correlations. I think an important insight for personality researchers would be whether personalityMap or SurveyBot3000 (or any other AI model) is better at predicting correlations in your study. Needless to say, I would also give this recommendation to Hommel & Arslan (2024) and suggest that they include PersonalityMap in a revision of their preprint.

Response: Thank you for this suggestion! We have followed it in full and have included SurveyBot3000 in our paper. Specifically, we have added an ‘Additional Results’ section to our paper where we discuss the results. Second, we have also added an appendix (Appendix D) where we detail the full results and comparisons with this model. However, there is a central drawback to the addition of this model, which is that SurveyBot3000 was trained on what is our test set (Hommel & Arslan 2024, p. 21). We hope to have made this clear in all parts of the paper.

Example: SurveyBot3000 (Hommel & Arslan 2024) is a fine-tuned model that uses the sentence transformer all-mpnet-base-v2 as the pre-trained model, that was then fine-tuned in two stages: polarity calibration and domain adaptation. Their pilot results showed strong accuracy in predicting empirical inter-item correlations, as well as scale reliabilities and inter-scale correlations. We tested SurveyBot3000’s performance also on our item pairs to

further contextualise the results of PersonalityMap and our set of LLMs. We find that SurveyBot3000 exceeds the performance of human experts in individual comparisons and is indistinguishable from them in aggregate analyses. While PersonalityMap's performance point estimates are superior to those of SurveyBot3000 on three of our performance measures (correlation, mean error, and win rate against experts), the differences are small and not statistically significant - making it appear that SurveyBot3000 is on par with PersonalityMap. However, the interpretation of these findings is very tentative since SurveyBot3000, in contrast to PersonalityMap, was trained on the SAPA inventory (Hommel & Arslan 2024, p. 21). This means that our test set was part of SurveyBot3000's training data, making direct comparisons to our models not possible since the reported performance here is compromised by data contamination (i.e., we are unable to tell if SurveyBot3000's performance is due to genuine generalisation ability, or simple memorization, since it was trained on the answers that we use to test the performance of all our methods). For completeness, we report SurveyBot3000's performance on our preregistered hypotheses in Appendix D.

Comment 4: Additional points: (4) The methodology section is missing some important information. What sample of participants were the correlation coefficients derived from? What was the population, what was the sample size? This would provide important context for the results, since imprecision or bias in estimating the true correlations places an upper bound on the performance that can be achieved.

Response: Thank you so much for pressing us on this, we definitely forgot to add this - referring now to Condon et al. (2017) we state clearly in the methods section that the total sample size was over 125,000 participants from more than 220 countries.

Example: For our test data set, we use 249 pairs of personality psychology items taken from the SAPA Personality Inventory (Condon et al. 2017). This inventory drew on a total of “125,000 study participants from over 220 countries or regions” (Condon et al. 2017) over the course of their exploratory, replication, and confirmatory samples.

Comment 5: (5) Id suggest calculating the ICC(2,k) https://doi.org/10.1037/0033-2909.86.2.420 as an estimate of the interrater reliability of the mean rating for a sample of k judges, which would give an indication of whether adding additional judges would increase reliability. For example, this statistic is implemented in R packages such as psych by William Revelle. Since this is not currently reported in the manuscript, I have no way to be sure that 3 runs are sufficient to achieve a sufficient reliability of, for example, ICC(2,k) > .95. If the ICC(2,k) turns out to be below that for GPT-4o or Claude 3, then more runs should be added to these methods to potentially improve their performance.

Response: Thank you very much for this great suggestion! In response reviewer comments from you and one other reviewer, we were able to also test this at a higher temperature version with more runs. We do find ICC(2,k)>0.95 for the low temperature runs on both GPT-4o and

Claude 3 Opus, though additional runs at a higher temperature lead to even higher reliability at 0.99.

Example: The addition of the high-temperature version of GPT-4o also allows us to test the interrater reliability of our LLMs, specifically between the low-temperature GPT-4o queries at three runs per item and the high-temperature GPT-4o queries at 30 runs per item. In exploratory analyses, we calculated the Intraclass Correlation Coefficient (ICC) using a two-way random-effects model to assess the consistency of the models' predictions across multiple runs. The ICC(2,1), representing the reliability of a single run, was higher for the low-temperature GPT-4o (ICC(2,1) = 0.874) compared to the high-temperature GPT-4o (ICC(2,1) = 0.826). This indicates that individual runs at low temperature produce more consistent predictions than individual runs at high temperature, as expected due to the increased randomness introduced by higher temperature settings. However, when considering the reliability of the average predictions across runs, the ICC(2,k) shows a different picture. The ICC(2,k) for the low-temperature GPT-4o (with k = 3 runs) was 0.954, while the ICC(2,k) for the high-temperature GPT-4o (with k = 30 runs) was 0.993. Despite the higher variability in individual runs at high temperature, the larger number of runs significantly enhances the reliability of the average prediction. This occurs because averaging over more runs reduces the impact of random errors, leading to a more stable and consistent aggregate prediction. Therefore, while high-temperature settings introduce more variability in single outputs, the aggregation of multiple runs yields highly reliable results, even surpassing the reliability of fewer low-temperature runs.

Comment 6: (6) I think that predicting the (relative) pattern of correlation coefficients (as done in H2b) is much more informative about true performance than predicting the absolute correlation coefficients (as done in H2a), especially given that the correlations were derived from self-report data and are unlikely to represent "true" correlations between different experiential or behavioral patterns. Self-reports are likely to be inflated by various sources of systematic measurement error that affect the general assessment of correlation estimates, such as biases related to global self-report (e.g., known as common method biases; Podsakoff et al., 2024 https://doi.org/10.1146/annurev-orgpsych-110721-040030). Note that correlations derived from multimethod data tend to be much lower. Thus, could the better performance in predicting absolute correlation coefficients as found in self-reports also be explained by the reproduction of design-specific biases associated with the use of self-reports (rather than true signal)? I suggest that this aspect be discussed more critically and that more weight be given to the results for the relative pattern of correlation coefficients in the conclusions, which suggest that the median ratings of laypersons and experts and the ratings by PersonalityMap seem to be on a par in terms of predictive performance.

Response: We appreciate the reviewer's insightful comment regarding the influence of self-report biases on predicting absolute correlation coefficients. In the original manuscript, we did try to point out that AI models’ advantages might be largey quantitative. For example, in our discussion of why we might get null results in the bucketized predictions analyses for

Hypothesis 2c, we began a paragraph with “This result suggests that much of the prediction advantage that a system like PersonalityMap has over human predictors lies in the ability to determine the size of correlations, rather than simply whether they are positive, negative, or close to zero.” But we appreciate that our original discussion didn’t place sufficient emphasis on the possibility that this quantitative advantage might result from accounting for survey response biases etc..

To address this in the revised manuscript, we have expanded the discussion to critically engage with how such biases may affect the accuracy of absolute predictions. We have also made changes to the conclusions at later stages of the paper throughout.

In addition, we now emphasise the practical utility of predicting correlations between personality items for purposes such as hypothesis testing and scale development, despite the presence of biases like common method bias. However, we recognise that these biases may have contributed to the observed performance in predicting absolute correlations.

Example: There are many reasons that it can be useful to understand the relationships between self-reported personality items, including to test hypotheses (e.g., that people with anxiety also often have depression), to develop scales (e.g., to identify items that could help measure a trait such as narcissism), and to generate new hypotheses (e.g., by exploring what items are predictive reporting being unhappy with their relationships). For these same reasons, it can be useful to accurately predict the correlations between personality items. Additionally, such predictions of correlations are much faster to make than running studies to measure those correlations, so sufficiently accurate correlation predictions could facilitate rapid research—allowing the data collection (to confirm those predictions on real people) to be pushed back later at the end of the research process.

One possibility for why AI systems may exceed human performance so much more in terms of absolute error than when predicting the correct buckets could be that AI systems model not just true underlying correlations between traits but also method factors or design-specific biases that can be associated with the use of self-reported responses - and these biases are much more impactful in terms of absolute error than in terms of predicting the correct bucket. On the other hand, if we interpret the prediction task as predicting what the actual measured correlation will be between items, then such biases are precisely part of what is intended to be predicted. While it is sometimes advantageous to calculate correlations between, for example, a self-reported personality question and an objectively measured item (e.g., income from pay stubs) to help reduce some biases, calculating correlations between self-reported items is still a commonly used procedure for a wide variety of purposes, and any such biases that exist in self-reporting will be captured in those correlations as well.

Comment 7: (7) In a similar vein, from a descriptive perspective, it would be useful to briefly describe the distribution of scores for the different methods and compare them to the distribution of correlation coefficients. Also, Figure 2 only shows the absolute prediction errors, not whether

the methods tend to over- or underestimate the correlations.

Response: Thank you very much for this suggestion! We initially had Figure 3 to answer this question, which plotted prediction errors by condition. However, as you point out, this does not allow for an analysis of over- or underestimation. To address this, we now reproduce Figure 3, but instead of using prediction error, we plot signed prediction error.

Reviewer #3

General Comment: The paper explores the ability of LLMs (GPT-4o and Claude 3 Opus) and a specialized model called PersonalityMap, to predict correlations between personality questionnaire items. The study compares these model-based predictions with those made by laypeople and academic experts. The findings reveal that while ML models, especially PersonalityMap, outperform most individual humans, aggregate expert predictions still match or surpass the performance of LLMs. While the topic is of scientific interest and the manuscript has the potential to make a novel contribution, there are several major issues that should be addressed prior to publication.

Response: Thank you very much for your actionable set of comments! We hope that the revised manuscript addresses all of them! In the revised paper, we also highlight in yellow the passages that were most heavily rewritten or had been added.

Comment 1: Major Concerns 1) Motivation and Integration with Prior Work

While the overall question about ML models' "understanding" of human personality is important, the authors boil this problem down to a much more narrow question about the prediction of correlations between questionnaire items. In doing so, the authors miss the opportunity to (1) sufficiently motivate their research and (2) tie their work in with prior research on the topic. Specifically, there is a whole stream of research investigating different aspects of LLM's understanding of personality, including (1) their ability to infer personality from text and conversational interactions, (2) simulate personality-congruent responses, and 3) utilize personality to adaptively interact with humans.

Relevant sources:
Peters, H., & Matz, S. (2024). Large language models can infer psychological dispositions of social media users. PNAS Nexus, pgae231. https://doi.org/10.1093/pnasnexus/pgae231

Zhang, Tianyi, Antonis Koutsoumpis, Janneke K. Oostrom, Djurre Holtrop, Sina Ghassemi, and Reinout E. de Vries. "Can Large Language Models Assess Personality from Asynchronous Video Interviews? A Comprehensive Evaluation of Validity, Reliability, Fairness, and Rating Patterns." IEEE Transactions on Affective Computing, 2024, 1–16. https://doi.org/10.1109/TAFFC.2024.3374875.

Peters, H., Cerf, M., & Matz, S. C. (2024). Large Language Models Can Infer Personality from Free-Form User Interactions (arXiv:2405.13052). arXiv. https://doi.org/10.48550/arXiv.2405.13052

Pellert, Max, Clemens M. Lechner, Claudia Wagner, Beatrice Rammstedt, and Markus Strohmaier. "AI Psychometrics: Assessing the Psychological Profiles of Large Language Models

Through Psychometric Inventories.” Perspectives on Psychological Science, January 2, 2024, 17456916231214460. https://doi.org/10.1177/17456916231214460.

Jiang, Guangyuan, Manjie Xu, Song-Chun Zhu, Wenjuan Han, Chi Zhang, and Yixin Zhu. “Evaluating and Inducing Personality in Pre-Trained Language Models.” Advances in Neural Information Processing Systems 36 (December 15, 2023): 10622–43.

Dorner, Florian E., Tom Sühr, Samira Samadi, and Augustin Kelava. “Do Personality Tests Generalize to Large Language Models?” arXiv:2311.05297 [Cs], November 9, 2023. https://doi.org/10.48550/arXiv.2311.05297.

Matz, S. C., Teeny, J. D., Vaid, S. S., Peters, H., Harari, G. M., & Cerf, M. (2024). The potential of generative AI for personalized persuasion at scale. Scientific Reports, 14(1), 4692. https://doi.org/10.1038/s41598-024-53755-0

I suggest the authors thoroughly review the previous literature and then motivate their own work by explaining how it adds to prior research in this field. Additionally, the authors should carefully justify why it is important to understand the relationships between personality items, what their research would reveal about the abilities of AI models, and what theoretical or practical implications their results could have.

Response: Thank you for pushing us on this! We have added the additional literature and have attempted to improve our motivation in the introduction section.

Example: Moreover, our work fits into a broader literature on LLMs and personality more generally. This work has investigated various aspects of how AI models understand and interact with human personality. For instance, Peters and Matz (2024) demonstrated that LLMs like GPT-3.5 and GPT-4 can accurately infer individuals' Big Five personality traits from social media posts, highlighting the potential of LLMs to analyze psychological dispositions. Zhang et al. (2024) evaluated LLMs' ability to assess personality from asynchronous video interviews, finding that while LLMs can achieve validity comparable to task-specific AI models, they exhibit uneven performance across different traits. Other research has focused on LLMs' capability to simulate personality-congruent responses and adaptively interact with humans (Peters et al. 2024; Matz et al. 2024), as well as assessed the psychological profiles of LLMs themselves through psychometric inventories (Pellert et al. 2024), and outlined concerns about the generalizability of personality tests to AI models (Sühr et al. 2023). Jiang et al. (2023) further explored inducing specific personality traits in pre-trained language models, demonstrating controlled and verifiable behavior changes.
Despite these advancements, there remains a gap in the field’s understanding: it is still unclear how well AI models comprehend and predict the underlying relationships between personality questionnaire items. Accurately predicting such correlations is useful for validating scales, testing hypotheses, and gaining deeper insights into the structure of personality traits. While previous research has focused on LLMs' ability to simulate personality-consistent behavior or infer traits from text, few studies have directly addressed their ability to predict the correlations

between individual personality items, which is central to psychometric research and practical research applications. By focusing on this specific task, our work fills a critical gap, showing how well frontier LLMs and specialised models can perform in comparison to human experts and laypeople.
There are many reasons that it can be useful to understand the relationships between self-reported personality items, including to test hypotheses (e.g., that people with anxiety also often have depression), to develop scales (e.g., to identify items that could help measure a trait such as narcissism), and to generate new hypotheses (e.g., by exploring what items are predictive reporting being unhappy with their relationships). For these same reasons, it can be useful to accurately predict the correlations between personality items. Additionally, such predictions of correlations are much faster to make than running studies to measure those correlations, so sufficiently accurate correlation predictions could facilitate rapid research—allowing the data collection (to confirm those predictions on real people) to be pushed back later at the end of the research process.
AI systems capable of reliably predicting these correlations could automate and streamline psychometric assessments, enhancing the development of personality scales and hypothesis testing. If general models like LLMs could achieve high accuracy, they might enable applications in hiring, healthcare, and personalized marketing, reducing reliance on extensive questionnaires or expert input while adapting quickly to new contexts.

Comment 2: 2) Justification of Hypotheses

The fact that the authors pre-registered their hypotheses is applaudable. However, since there is no theoretical grounding or rationale given for the hypotheses the research still strikes me as, overall, exploratory in nature. The authors should attempt to justify their hypotheses more thoroughly.

Response: Thank you for pointing this out! We indeed did not sufficiently motivate the hypotheses at this point, which we have now addressed throughout the Hypotheses section.

Example: Our first research question is about how machine learning model approaches perform in the distribution of individual human predictions. This research question is important primarily for a head-to-head comparison between AI models and humans. For example, in academic research one may want to draw on correlations between items to help theory-building or experiment design. When these relationships are not yet studied (or may be the object of the study in question), researchers may use expert predictions as a stand-in for early hypothesis generation. Our individual analyses query whether models could be used instead of humans for tasks like this. To test this specifically, we approach it in two ways. First, we test whether the machine learning model approaches have a better or worse average error (over all predicted items) than the median lay person and the median expert (ranked based on the average error for the subset of questions they answered).

Our second set of questions, aimed at our second research question, compares the accuracy of all the approaches by taking the median prediction for each item within each condition before comparison to arrive at an aggregate prediction. This set of analyses aims to give the AI approaches a tougher comparison to humans. While AI models might beat individual humans, even experts, aggregated human forecasts may prove a more difficult challenge as they draw on the distributed knowledge that a diverse group of humans inevitably possesses. In a sense, this allows us to test not whether individual AI queries can replace queries to individual experts, but whether these systems may be used as full stand-alone replacements to human expert consensu ses too. After all, if even aggregations of expert opinions cannot provide better estimations of relationships, this might open up a whole host of applications across industries. To test whether this is indeed the case, we analyse the differences in prediction error, prediction correlation, and bucketised prediction error between the conditions. These individual scores indicate how well the different approaches work as an aggregate.

Comment 3: 3) Variability in Model Outputs and Wisdom of Crowds Effects

In the current setup, summary statistics are not very meaningful for ML models, given the low number of LLM runs and the decision to use a temperature of T=0, which reduces the variability in outputs. Therefore, Table 1 does not seem to add much to the overall story of the manuscript. More importantly, the findings regarding the relative accuracy of median human estimates beg the question as to whether a similar effect would be observed for LLM-inferred scores. I suggest the authors run an additional experiment where LLMs repeatedly generate scores over a larger number of iterations with a higher temperature value (e.g., the default of T=1), and analyze how aggregate scores compare to human aggregates and PersonalityMap.

Response: We appreciate your criticism of Table 1. To address it, we now also clarify the implications of T=0 in the text around Table 1.

On your second point: Thank you for suggesting this! We have followed your recommendation (which was also made by Reviewer 2) and have added an additional condition for GPT-4o that runs at T=1 (at 30 queries per item). Budgetary constraints have made it not feasible to do the same for Claude 3 Opus. We find that for the most part, the high temperature model is not statistically different from the low temperature runs. We do find one exception though, where despite the high temperature results remaining statistically indistinguishable from the low temperature runs, significant differences do arise with respect to other conditions (this is specifically the case for Null Hypothesis 2b). We have added this result to the ‘Additional Results’ section as well as a full analysis of all tests in Appendix D.

Example: Second, we also ran an additional condition with the same GPT-4o model that we preregistered (gpt-4o-2024-05-13), but at a high temperature (of 1) with 30 runs for each item (which we then take the median of), to further test current frontier model performance at different parameters. Temperature is a hyperparameter for LLMs that controls the randomness of the model's predictions - at higher temperatures models are more "creative", producing more

unique and unusual responses. While higher temperatures typically produce worse performance, one strategy to try to improve LLM performance is to generate more responses at higher temperature and then combine them (e.g., through taking the median). Of course, this also comes at greatly increased cost, since the LLM must be run many times. We find that for most of our preregistered comparisons, the high-temperature version of GPT-4o with 30 runs is on par with the low-temperature version at 3 runs. However, we find that in the aggregate analysis, drawing on the more varied predictions from higher temperature leads to improved performance on one of our measures. Specifically, with respect to the correlation between median predicted correlation (across the different runs of the same model) and the empirical correlation, the high-temperature multiple-runs condition of GPT-4o is not statistically different from PersonalityMap, whereas the low-temperature version of GPT-4o was statistically worse. For a full set of results of this model on our preregistered hypotheses, see Appendix D.

Comment 4: 4) Discussion Section

The discussion session is lacking in several ways. First, the discussion mostly restates the results but does not sufficiently tie the current research in with previous work and the broader literature. Second, the manuscript ends very abruptly with a very short Limitations section. I recommend expanding the Limitations section with material currently placed in footnotes, as well as concerns raised by reviewers. Finally, a separate section on practical implications and a short conclusion section would round out the paper.

Response: Thank you for these three points! We address them in turn:

a) Discussion section: We have spent more space discussing some relevant other work in our Discussion section (as well as the Limitations section).

b) Limitations section: We have massively expanded the Limitations section based on your and other reviewer's suggestion. The section is now more than one page, going through generalisability, incentivisation, and other potential concerns.

c) Practical Implications/Conclusions sections: We have now added short Practical Implications and Conclusions sections after the Limitations section as requested.

Examples: This result is consistent with findings from Peters and Matz (2024), who found that LLMs could accurately infer Big Five personality traits from social media data or free-form user interactions (Peters et al. 2024), suggesting that these models are adept at interpreting human psychological indicators across a number of contexts. Our evidence thus fits with previous work and reinforces the broader trend of machine learning models becoming adept at human personality tasks, sometimes reaching human expert performance. These findings might open up potential applications of machine approaches, both for general LLMs but especially for targeted ones like PersonalityMap and SurveyBot3000, with the former also being able to facilitate a back-and-forth between the human and the model via the now well-known chat bot

settings in which LLMs are often encountered, see Appendix B, and with the latter able to provide accurate predictions that may help expert applications.

One potential limitation of our study is that the data used, the SAPA Personality Inventory (Condon et al. 2017), may be part of the training data for both LLMs, GPT-4o and Claude 3 Opus. While we can be sure that this is not the case for PersonalityMap, as the studied item pairs were not part of the training data, it is possible that they have been part of the LLM training data which may thus overstate their ability to predict correlations between the sets of personality items. However, we want to point out that we were not able to find these individual correlations in the public domain, making it at least relatively plausible that they were not part of the training data. Additionally, for model results like SurveyBot3000, this limitation is more severe as it was trained on what ended up being our test set.

Another limitations is that our results are also limited by the choice of data we use to test the models and humans on, reducing generalisability. Specifically, we only use a single source to draw our item pairs from (the SAPA Inventory), resulting in a single item type of self-descriptive statements based on self-report in only one mode of assessment (cross-sectional). A further constraint on the generalisability of our findings is that we drew on a W.E.I.R.D. sample (Henrich et al. 2010, Simons et al. 2017). These concerns limit how much we can generalise from our results. It is plausible that different items might lead to different results that may not replicate in different samples. However, as self-report items remain the backbone of contemporary personality psychology, we believe that, at least with respect to the current paradigm, our results should hold up.

Another limitation is that our results are also limited by the choice of data we use to test the models and humans on, reducing generalisability. Specifically, we only use a single source to draw our item pairs from (the SAPA Inventory), resulting in a single item type of self-descriptive statements based on self-report in only one mode of assessment (cross-sectional). A further constraint on the generalisability of our findings is that we drew on a W.E.I.R.D. sample (Henrich et al. 2010, Simons et al. 2017). These concerns limit how much we can generalise from our results. It is plausible that different items might lead to different results that may not replicate in different samples. However, as self-report items remain the backbone of contemporary personality psychology, we believe that, at least with respect to the current paradigm, our results should hold up.

A third limitation is that we did not incentivise either of our human condition’s responses for accuracy. This may reduce their performance, which is something that future research can test.

Fourth, given that prediction questions were randomly assigned to humans, some correlations would be displayed to humans more than to machines and some less. While such differences would reduce the precision of our estimates of relative difference between human and AI approaches, since they would be random, this added noise is accounted for in our confidence intervals and significance tests

The results of this study highlight several potential practical implications for both psychometric research and applied fields such as human resources, healthcare, and marketing. Specialised AI models like PersonalityMap might be able to greatly expedite the research process by reliably

predicting personality trait correlations, facilitating faster hypothesis testing and scale development at a lower cost. AI might also be able to also assist in automating personality assessments, reducing the need for expert input in contexts such as hiring and diagnostics. However, while generalised LLMs offer broad applicability, they still seem less effective than domain-specific models in tasks requiring high accuracy.

Comment 5: Minor Concerns 5) “Our samples were willing to answer more questions than anticipated” (p. 9) - I assume you meant to say “participants”.

Response: Fixed!

Comment 6: 6) Throughout the manuscript the authors frequently refer to ML modes as “machines” in contrast to “humans”. This dichotomy has a pop-cultural connotation that the authors might want to avoid. Why not call them “ML models”?

Response: We see your point, and think that these two terms are equivalent in the way that we use it. Moreover, we use the term ‘model’ at twice the rate than the term ‘machine’. Additionally, having preregistered the ‘machine’ term, it would be quite difficult to replace all such instances, as we sometimes want to describe our preregistered hypotheses verbatim. Still, to address your point, we have removed more instances of ‘machine’ with ‘model’, though have not done so across the board for the reasons above.

Comment 7: 7) The footnote on page 14 is rather long and a bit confusing. Maybe it would make sense to add some context and discuss these points in the Limitations section?

Response: Good idea! We have now moved it.

Comment 8: 8) “However, it is also possible that, by a fluke of what items the humans were randomly assigned to predict, some item correlations might arise more in the human sample than in the machine sample” (p.14) - I assume you could verify empirically whether this is indeed the case.

Response: This is a great point, we have added this possibility to potential limitations.

Comment 9: 9) Tables should be understandable in isolation. Please make sure to include sufficient information in the notes. For example, it was not clear in Table 5 what the correlations were referring to.

Response: We have made the sets of table descriptions that deal with aggregated analyses more descriptive, making clear that it is the aggregated (i.e., median) predictions that are used.

Comment 10: 10) Why were CIs computed on z scores and not on the correlation coefficients themselves in Table 5? The latter seems more common and more intuitive. Relatedly, Figure 5 would be much more intuitive if it showed correlation coefficients instead of z scores.

Response: We preregistered our analysis using Fisher's z-scores to account for what we anticipated would be many conditions with similarly high correlations (e.g., 0.9 and above). In cases like these, z-scores offer a key advantage because the z-transformation stabilises the variance of correlation coefficients, particularly in the upper and lower extremes where correlations approach 1 or -1. This stabilisation enables us to compute confidence intervals (CIs) more accurately and ensures that the CIs remain symmetric, which is harder to achieve when working directly with raw correlations due to their potential skewness. While we understand that this may be less intuitive, we also report the full correlation values in Table 5.

Comment 11: 11) The analysis on bucketed prediction errors (Hypothesis 2c) does not seem to add much above and beyond the previous analyses. The authors might want to consider moving it to the SI.

Response: Thank you for the suggestion! We decided to keep this analysis in the main manuscript partly because this was our intention in preregistering it, but also because we think it does provide some useful insight. For example, the null results in bucketized predictions are suggestive evidence that the advantages of AI approaches stem primarily from the ability to quantify the size of correlations more precisely rather than from the ability to intuitive the qualitative direction of predictions. The prediction-correlation analyses for Hypothesis 2b suggest a similar conclusion, but we nonetheless think the bucketized predictions lend greater credence to it.

Comment 12: 12) "Our results suggest that current AI models are roughly as good as, if not better, than human experts in predicting correlations amongst human personality traits." (p. 25) - The wording is imprecise. The predictions are not about relationships between traits but between questionnaire items. A similar concern applies to the same statement on page 3.

Response: Thank you so much for pushing us on this! We had phrased this in a confusing way, as such differences are actually inevitable given how we set up the experiment, with AI answering all questions at a specific number and humans doing so with an element of randomness and drop out. We have rephrased this now.

Example: Fourth, given that prediction questions were randomly assigned to humans, some correlations would be displayed to humans more than to machines and some less. While such

differences would reduce the precision of our estimates of relative difference between human and AI approaches, since they would be random, this added noise is accounted for in our confidence intervals and significance tests

Comment 13: 13) “To make an analogy, imagine if biologists could only ever conduct research in human bodies (in vivo) without the ability to do experiments in vitro.” (p. 28) - This analogy confuses more than it clarifies.

Response: We have updated the analogy!

Example: Artificial intelligence systems like PersonalityMap, GPT-4o, or open source LLMs (cf Hussain et al., 2024), which can outperform even human experts on some measures of predicting psychological facts about humans, might open an intriguing possibility for the future of social science research. To make an analogy, imagine if biologists could only ever conduct research in human bodies (in vivo) without the ability to do experiments in vitro. In such cases, research in biology would be slowed tremendously. Test tube experiments allow for much faster iteration than is possible with direct human experiments (though of course, preliminary test tube results must ultimately be confirmed in humans). But no such in vitro approach to psychology experimentation has existed, until now. Systems like PersonalityMap and other AI based predictors might be able to act as a "digital test tube" where research can be rapidly performed and iterated. With such technologies, it may be possible for researchers to generate new hypotheses and conduct preliminary tests of hypotheses before conducting a single human experiment, which may accelerate the speed of research. For example, Manning, Zhu, & Horton (2024) used LLMs in combination with structural causal models to automate the generation, simulation, and testing of social science hypotheses by structuring experiments in scenarios like negotiations and auctions, where the system predicted causal relationships and tested them through in silico simulations. Of course, as with biological experiments in test tubes, before the research is finished, the findings must be confirmed in real humans to make sure they apply. Still, if early pilot studies could be replaced with queries to machine learning algorithms, it seems possible that the research process would be accelerated.

Comment 14: Conclusion
While the manuscript reveals novel insights on the ability of ML models to represent personality constructs, there are several major issues that need to be addressed. Most importantly, the authors need to better motivate their research question and integrate their work with the existing literature. The authors should also provide justifications for their hypotheses and conduct additional experiments to examine the quality of aggregate model-based predictions. Finally, the discussion section should be expanded to tie the findings back to the prior literature, address limitations more comprehensively, and outline the practical implications of the study. Additional revisions to the manuscript’s language, clarity, and table presentation would be helpful as well.

Given these substantial concerns, I recommend a major revision of the manuscript.

Response: Thank you very much for giving us the chance to revise our manuscript in light of your comments. We hope to have addressed all of them!
